# Genetic mutation and biological pathway prediction based on whole slide images in breast carcinoma using deep learning

**DOI:** 10.1038/s41698-021-00225-9

**Published:** 2021-09-23

**Authors:** Hui Qu, Mu Zhou, Zhennan Yan, He Wang, Vinod K. Rustgi, Shaoting Zhang, Olivier Gevaert, Dimitris N. Metaxas

**Affiliations:** 1 Department of Computer Science, Rutgers University, Piscataway, NJ USA; 2 Sensebrain Research, Princeton, NJ USA; 3 School of Medicine, Yale University, New Haven, CT USA; 4 Department of Medicine, Rutgers Robert Wood Johnson Medical School, New Brunswick, NJ USA; 5 SenseTime Research and Shanghai AI Laboratory, Shanghai, China; 6 Stanford Center for Biomedical Informatics Research (BMIR), Department of Medicine, Stanford University, Stanford, CA USA

**Keywords:** Cancer genomics, Breast cancer, Cancer imaging

## Abstract

Breast carcinoma is the most common cancer among women worldwide that consists of a heterogeneous group of subtype diseases. The whole-slide images (WSIs) can capture the cell-level heterogeneity, and are routinely used for cancer diagnosis by pathologists. However, key driver genetic mutations related to targeted therapies are identified by genomic analysis like high-throughput molecular profiling. In this study, we develop a deep-learning model to predict the genetic mutations and biological pathway activities directly from WSIs. Our study offers unique insights into WSI visual interactions between mutation and its related pathway, enabling a head-to-head comparison to reinforce our major findings. Using the histopathology images from the Genomic Data Commons Database, our model can predict the point mutations of six important genes (AUC 0.68–0.85) and copy number alteration of another six genes (AUC 0.69–0.79). Additionally, the trained models can predict the activities of three out of ten canonical pathways (AUC 0.65–0.79). Next, we visualized the weight maps of tumor tiles in WSI to understand the decision-making process of deep-learning models via a self-attention mechanism. We further validated our models on liver and lung cancers that are related to metastatic breast cancer. Our results provide insights into the association between pathological image features, molecular outcomes, and targeted therapies for breast cancer patients.

## Introduction

Breast carcinoma is the most common cancer among women worldwide that consists of a heterogeneous group of diseases with different histological, prognostic, and clinical outcomes^1^. Approximately 50% of all women diagnosed with breast cancer can develop metastatic diseases, such as liver and lung cancers^2^. In the past decades, substantial efforts have been made to deepen our understanding of breast cancer risk factors, molecular pathogenesis, and treatment development. Especially, high-throughput molecular profiling reveals that multiple genetic mutations and biological signaling pathways could have a great influence on tumor progression and overall survival^3^.

Comprehensive genomic analysis has identified key driver genetic mutations that are responsible for therapeutic implication and outcome prediction of breast cancer. The tumor suppressor gene TP53 is found altered in breast carcinoma in ~30% of all cases with prognostic implication^4^. Overexpression of ERBB2 is also an adverse prognostic indicator correlated with decreased survival in breast cancer^5^. Given certain types of mutations, targeted therapies for patient subgroups have been developed. For example, the PI3K inhibitor is designed to be responsive for patients with the PIK3CA mutation, which is a key driver gene associated with oncogenesis and hyperactivity of the PI3K pathway. The identification of driver mutations is essential for targeted therapy and clinical diagnosis of breast malignancies.

Digital whole-slide images (WSI) can potentially offer a computationally effective and efficient means to quantitatively characterize cell-level heterogeneity of cancer specimens. Pathologists routinely use WSIs to identify nuclei features, diagnose cancer status and measure the histopathological grade of cancer tissues. However, there is a lack of research linking WSI with gene mutations and pathway activities for advancing clinical assessment in breast cancer. Preliminary evidence suggests that it is possible to apply deep-learning approaches to automatically predict cancer subtypes in multiple cancers^6–8^, predict mutations in lung^6^ and liver cancers^9^, classify mesothelioma^10^, detect DNA methylation patterns^11^, estimate human epidermal growth factor receptor 2 status in breast cancer^12^, and predict pan-cancer prognosis for patients^13^. However, pan-cancer studies^14–16^ are unable to provide deep characterization of breast cancer across histopathology, mutation, and pathway activity levels.

In this study, we develop WSI-based deep-learning classifiers for predicting key mutation outcomes and important biological pathway activities in breast cancer. We directly provide slide-level predictions with a self-attention mechanism. This self-attention technique can capture the relationship between patches and empower us to visualize representative tiles during the decision-making process. Our study highlights WSI visual interactions between mutation and its related pathway, enabling a head-to-head comparison to reinforce our major findings. Furthermore, we validate our analysis in a pan-cancer setting on liver and lung cancer cohorts to gain additional insights of mutation prediction across cancers based on the metastatic associations derived from breast cancer.

## Results

### Datasets

We collected 659 patients with breast invasive carcinoma from The Cancer Genome Atlas (TCGA)^17^. Data inclusion criteria for each patient contain: (1) one hematoxylin and eosin (H&E) stained histopathology whole-slide image (WSI), (2) mutational data with the point mutation status of 18 driver genes and copy number alteration (CNA) of 35 genes (see Methods), and (3) omics data with the mRNA expression data and CNA data of all genes. The WSI data were downloaded from Genomic Data Commons data portal (https://portal.gdc.cancer.gov/) and the other molecular data were obtained from the cBioPortal (https://www.cbioportal.org/). The 659 cases in the TCGA-BRCA dataset were randomly partitioned into training, validation and test sets based on 70%, 15%, and 15% ratios, respectively. The characteristics of the breast cancer patients in each set are shown in Table 1. In addition, we collected 350 patients with lung adenocarcinoma from TCGA-LUAD cohort and 316 patients with liver hepatocellular carcinoma from TCGA-LIHC cohort to validate our method following the same data inclusion criteria. Each dataset is randomly split into training and testing sets with 20 and 80% ratios, where the training set is utilized to fine-tune the developed models trained from the breast cancer data. The patients’ characteristics of the lung and liver datasets are shown in Table 2. After WSI tile extraction and tumor tile selection (see Methods), there are 703,804, 140,981, and 167,530 tiles used in the training, validation and testing sets of breast cancer, 130,659 and 465,925 tiles in the training and testing sets of lung cancer, and 116,635 and 570,073 tiles in the training and testing sets of liver cancer.

**Table 1:** Patient characteristics on the 659 cases from TCGA-BRCA cohort.

	TCGA-BRCA			
	Train (*n* = 461)	Val (*n* = 99)	Test (*n* = 99)	Overall (*n* = 659)
Age (year)				
Average	56.6	57.6	57.4	56.9
Range	27–90	26–90	34–90	26–90
Sex, *n* (%)				
Male	7 (1.5)	3 (3.0)	0 (0.0)	10 (1.5)
Female	454 (98.5)	96 (97.0)	99 (100.0)	649 (98.5)
Stages, *n* (%)				
I/IA/IB	78 (16.9)	14 (14.1)	18 (18.2)	110 (16.7)
II/IIA/IIB	273 (59.2)	56 (56.6)	56 (56.6)	385 (58.4)
III/IIIA/IIIB/IIIC	95 (20.6)	25 (25.3)	24 (24.2)	144 (21.9)
IV	8 (1.7)	2 (2.0)	0 (0.0)	10 (1.5)
X	3 (0.7)	2 (2.0)	1 (1.0)	6 (0.9)
N/A	4 (0.9)	0 (0.0)	0 (0.0)	4 (0.6)
Subtypes, *n* (%)				
Luminal A	186 (40.3)	47 (47.5)	44 (44.4)	277 (42.0)
Luminal B	105 (22.8)	17 (17.2)	20 (20.2)	142 (21.5)
Her2	34 (7.4)	6 (6.1)	5 (5.1)	45 (6.8)
Basal	91 (19.7)	13 (13.1)	20 (20.2)	124 (18.8)
Normal	14 (3.0)	5 (5.1)	3 (3.0)	22 (3.3)
N/A	31 (6.7)	11 (11.1)	7 (7.1)	49 (7.4)

**Table 2:** Patient characteristics on the 350 cases from TCGA-LUAD cohort and 316 cases from TCGA-LIHC cohort.

	TCGA-LUAD			TCGA-LIHC		
	Train (*n* = 70)	Test (*n* = 280)	Overall (*n* = 350)	Train (*n* = 63)	Test (*n* = 253)	Overall (*n* = 316)
Age (year)						
Average	64.6	64.9	64.9	59.7	59.3	59.4
Range	40–85	38–88	38–88	17–84	16–90	16–90
Sex, *n* (%)						
Male	37 (52.9)	119 (42.5)	156 (44.6)	38 (60.3)	174 (68.8)	212 (67.1)
Female	33 (47.1)	161 (57.5)	194 (55.4)	25 (39.7)	79 (31.2)	104 (32.9)
Stages, *n* (%)						
I/IA/IB	40 (57.1)	154 (55.0)	194 (55.4)	30 (47.6)	120 (47.4)	150 (47.5)
II/IIA/IIB	14 (20.0)	76 (27.1)	90 (25.7)	12 (19.0)	63 (24.9)	75 (23.7)
III/IIIA/IIIB/IIIC	8 (11.4)	37 (13.2)	45 (12.9)	13 (20.6)	54 (21.3)	67 (21.2)
IV	7 (10.0)	12 (4.3)	19 (5.4)	0 (0.0)	3 (1.2)	3 (0.9)
X	0 (0.0)	0 (0.0)	0 (0.0)	0 (0.0	0 (0.0)	0 (0.0)
N/A	1 (1.4)	1 (0.4)	2 (0.6)	8 (12.7)	11 (4.3)	19 (6.0)

### Prediction of gene mutation status in breast cancer from pathology images

**Fig. 1:**
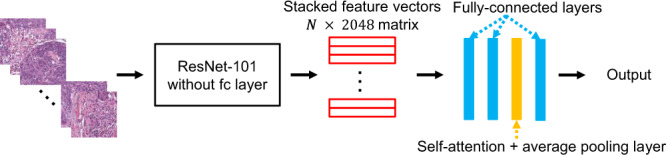
The proposed network structure. Each color normalized tile is fed into a pretrained ResNet-101 to extract a 2048-dimensional feature vector. Feature vectors of all tiles of the same patient are stacked and fed into a MLP with self-attention to predict the mutation probability or pathway activity.

**Fig. 2:**
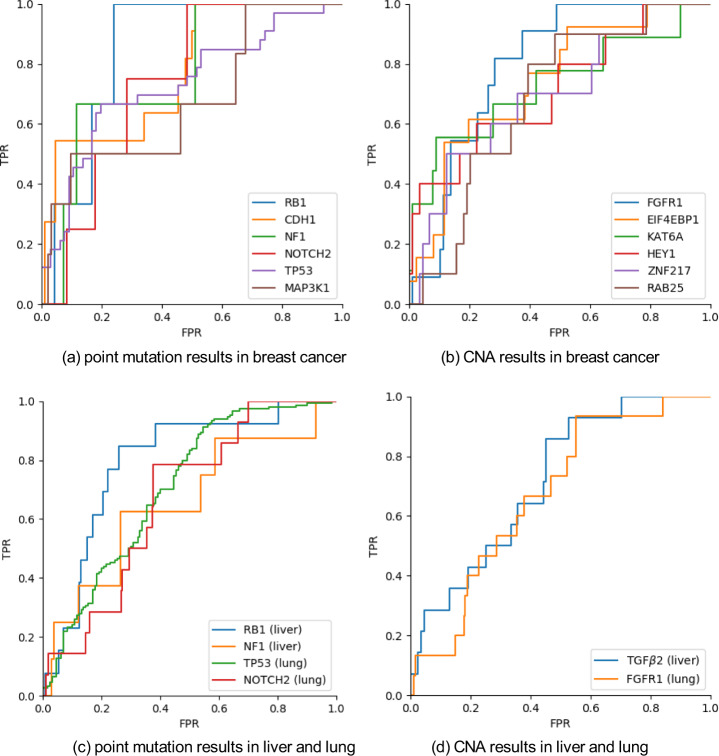
ROC curves of the top prediction outcomes. Results include (**a**) top point mutations and (**b**) top CNA predictions in breast cancer. Also (**c**) and (**d**) provide the associated validation results on point mutation and CNA predictions respectively.

We trained our models on pathology images to predict significant mutations profiles in breast cancer. More specifically, our deep-learning model extracted mutation-specific feature vectors from tumor tiles and predicted the gene mutation probability of the corresponding patient (see Fig. 1 and Methods). We seek to predict two types of gene mutations including point mutation and CNA. Our model demonstrated high-level performance on predicting the point mutation status of multiple important genes. The AUC scores for point mutation and CNA are shown in Table 3 and Table 4, respectively, and the corresponding ROC curves are shown in Fig. 2. For example, we found that our model is highly predictive on TP53 (AUC = 0.729), which is the most frequently mutated gene in breast cancer with prognostic implication. Our models also showed good results on predicting mutations of RB1 (AUC 0.852), CDH1 (AUC 0.776), NF1 (AUC 0.768), NOTCH2 (AUC 0.740) in breast cancer.

**Table 3:** AUC (with 95% CI) achieved by the models trained on the point mutation data of breast cancer.

Gene	RB1	CDH1	NF1	NOTCH2	TP53	MAP3K1
AUC	0.852 (0.740–0.969)	0.776 (0.625–0.914)	0.768 (0.449–0.949)	0.740 (0.515–0.917)	0.729 (0.621–0.828)	0.682 (0.419–0.949)

The top 6 results are reported out of 18 genes.

**Table 4:** AUC (with 95% CI) achieved by the models trained on the copy number alteration (CNA) data of breast cancer.

Gene	FGFR1	EIF4EBP1	KAT6A	HEY1	ZNF217	RAB25
AUC	0.794 (0.677–0.894)	0.742 (0.595–0.871)	0.732 (0.523–0.941)	0.715 (0.510–0.894)	0.693 (0.498–0.870)	0.686 (0.528–0.826)

The top 6 results are reported out of 35 genes.

**Fig. 3:**
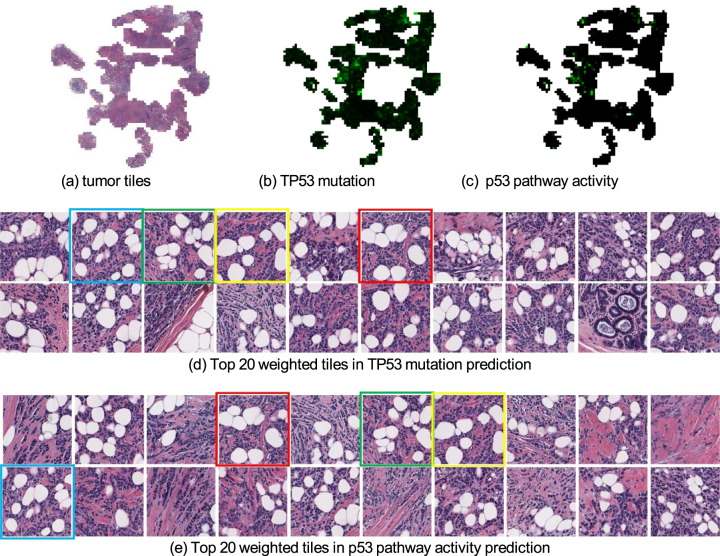
Weight maps of tiles when predicting the point mutation status of TP53 and p53 pathway activity from mRNA expression data in breast cancer. **a** Tumor tiles after data processing. **b** Weight map of tumor tiles in TP53 point mutation prediction. Brighter green tiles have larger weights. **c** Weight map of p53 pathway activity prediction. **d,** **e** Top 20 weighted tiles for TP53 point mutation prediction and p53 pathway prediction, respectively. We marked four tiles that appear in both tasks. These tissues contain poorly differentiated breast carcinoma with small nests, solid sheets, and single cells from a pathologist’s perspective.

**Fig. 4:**
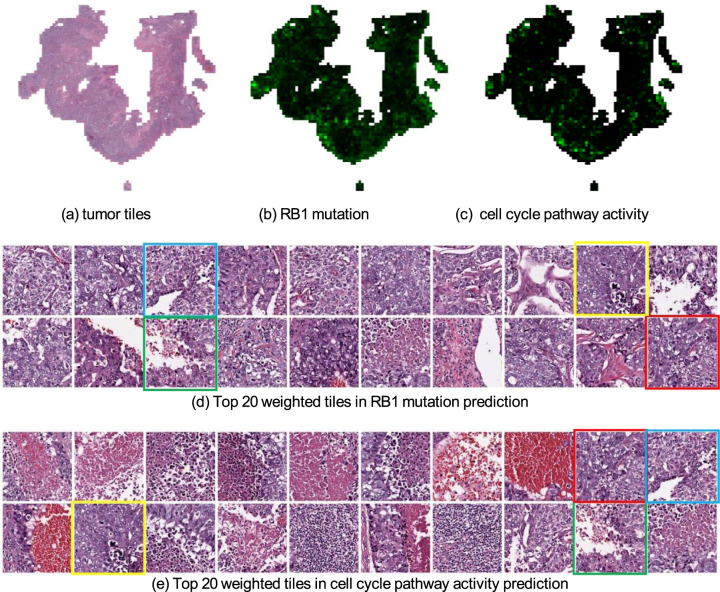
Weight maps of tiles when predicting the point mutation status of RB1 and cell cycle pathway activity from mRNA expression data in breast cancer. **a** Tumor tiles after data processing. **b** Weight map of tumor tiles in RB1 point mutation prediction. Brighter green tiles have larger weights. **c** Weight map of the cell cycle pathway activity prediction. **d,** **e** Top 20 weighted tiles for the RB1 point mutation prediction and cell cycle pathway prediction, respectively. We marked four tiles that appear in both tasks. These tissues contain poorly differentiated breast carcinoma with necrosis or hemorrhage from a pathologist’s perspective.

We also found that our deep-learning classifier predicted well (AUC > 0.65) on the CNA status in breast cancer, including six genes of FGFR1, EIF4EBP1, KAT6A, HEY1, ZNF217, and RAB25. More importantly, the use of the self-attention mechanism makes our deep-learning approach explainable, which enabled us to identify key tiles in the process of model prediction (Fig. 3). For example, we computed each tile’s weight that contributes to the final global feature vector and presented the weight map of a patient for TP53 in Fig. 3b and RB1 in Fig. 4b. The corresponding top 20 weighted tiles are also shown in Figs. 3d and 4d, respectively. Additional results of gene point mutation and CNA predictions can be found in Supplementary Table 1, Supplementary Table 2a, and Supplementary Table 2b.

### Prediction of biological pathway activity from histopathology images

We developed deep-learning models to predict the activities of ten canonical biological pathways^18^ identified in breast cancer for each patient. The pathway activity levels were derived from either mRNA expression data or the CNA data (see Methods) to supervise the model training. The model structure and training method were kept the same as in the mutation prediction task.

When using the mRNA expression data to represent pathway activity, we found that the p53, PI3K, and cell cycle pathways are predictable (AUC > 0.65, Table 4). When using the CNA data for pathway activity, the Myc pathway achieves the highest AUC (0.7950). The notch pathway (AUC 0.668) and p53 pathway (AUC 0.640) also have significant performance. Additional results of pathway activity predictions can be found in Supplementary Table 3 and Supplementary Table 4.

We also explored the visual interpretation of biological pathway activity and driver mutations as shown in histopathology images. To allow a joint analysis, we chose the biological pathway^18^ that is associated with the available driver gene mutations. For example, the visualization of the weight map on the p53 pathway is shown in Fig. 3c and the top 20 weighted tiles are also offered in Fig. 3e. Meanwhile, we used the same patient as the result in gene mutation prediction (Fig. 3), which shows the highlighted areas in TP53 mutation prediction (Fig. 3b) and the top 20 weighted tiles (Fig. 3d). We found that those tiles are highly correlated to both TP53 mutation and p53 pathway activity. This finding increases the confidence in our prediction because TP53 is a key gene in the p53 pathway, and one would expect a relationship between the mutation status and pathway activity (Tables 5, 6). Similarly, the same observation can be found between RB1 mutation prediction result and cell cycle pathway prediction result in Fig. 4. Additional results can be found from Supplementary Fig. 1 to Supplementary Fig. 10. Overall, we have seen a shared similarity among highlighted tiles despite the complexity of biological pathway activities.

**Table 5:** AUC (with 95% CI) achieved by the models trained on the pathway activity derived from mRNA expression data of breast cancer.

Pathway	p53	PI3K	Cell cycle
AUC	0.798 (0.696–0.890)	0.666 (0.544–0.777)	0.654 (0.543–0.760)

The top 3 results are reported out of 10 canonical pathways.

**Table 6:** AUC (with 95% CI) achieved by the models trained on the pathway activity derived from copy number alteration data of breast cancer.

Pathway	Myc	Notch	p53
AUC	0.795 (0.671–0.893)	0.668 (0.536–0.795)	0.640 (0.344–0.939)

The top 3 results are reported out of 10 canonical pathways.

### Validation of our deep-learning model on lung and liver cancers

Next, we validated our modeling approach in two different cancers namely lung adenocarcinoma and hepatocellular carcinoma. In the lung adenocarcinoma (TCGA-LUAD) cohort, 9 genes for point mutation and 14 genes for CNA were used for model testing (see Methods). Our fine-tuned models developed from the breast cancer cohort can predict the point mutation of TP53 (AUC 0.705) and Notch2 (AUC 0.656), the copy number alteration of FGFR1 (AUC 0.676), the p53 pathway activity (AUC 0.602) from mRNA expression data, and the activities of Myc pathway (AUC 0.658) and PI3K pathway (AUC 0.601) from CNA data. Overall, the responses on the pathway prediction are not as good as those on mutation prediction. Notably, the mutations of TP53 gene occur in about 50% of non-small cell lung cancer (NSCLC) and TP53 mutation is associated with worse prognosis with treatment resistance^19^, therefore the prediction of TP53 mutation is also helpful for the diagnosis of lung cancer.

In the liver hepatocellular carcinoma (TCGA-LIHC) cohort, the numbers of tested genes are 7 and 25 for point mutation and CNA, respectively. Our fine-tuned models can predict the point mutation of RB1 (AUC 0.795), the copy number alteration of TGFβ2 (AUC 0.718), the pathway activity of cell cycle from mRNA expression data (AUC 0.614), and Myc pathway activity from CNA data (AUC 0.602). In particular, RB1 is a key inhibitor of cell cycle progression in HCC patients^20–22^, and RB1 mutations are significantly associated with reduced cancer-specific and recurrence-free survival after resection in HCC patients^20–22^. Therefore, the prediction of RB1 mutation has potential prognosis value for those patients.

**Fig. 5:**
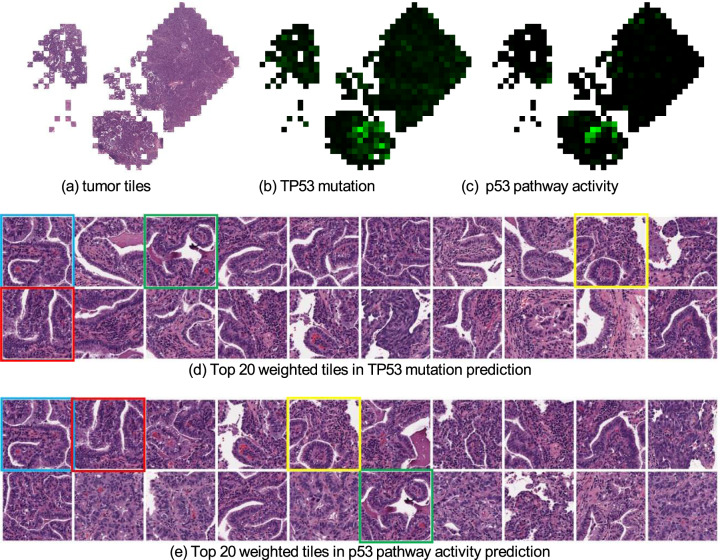
Weight maps of tiles when predicting the point mutation status of TP53 and p53 pathway activity from mRNA expression data in lung cancer. **a** Tumor tiles after data processing. **b** Weight map of tumor tiles in TP53 point mutation prediction. Brighter green tiles have larger weights. **c** Weight map in p53 pathway activity prediction. **d, e** Top 20 weighted tiles for the TP53 point mutation prediction and p53 pathway prediction, respectively. We marked four tiles that appeared in both tasks. These tissues contain moderately differentiated lung carcinoma with papillary growth pattern from a pathologist’s perspective.

**Fig. 6:**
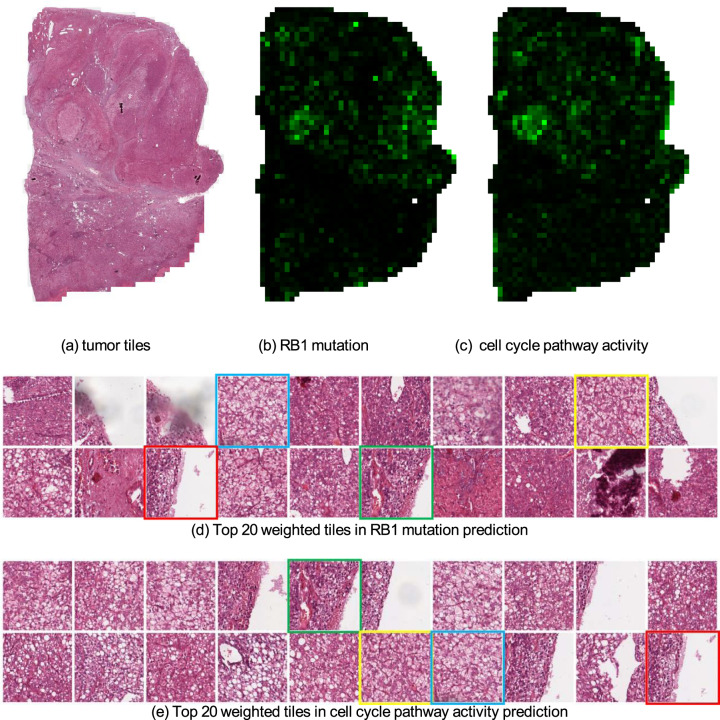
Weight maps of tiles when predicting the point mutation status of RB1 and cell cycle pathway activity from mRNA expression data in liver cancer. **a** Tumor tiles after data processing. **b** Weight map of tumor tiles in RB1 point mutation prediction. Brighter green tiles have larger weights. **c** Weight map in cell cycle pathway activity prediction. **d, e** Top 20 weighted tiles from RB1 point mutation prediction and cell cycle pathway prediction, respectively. We marked four tiles that appeared in both tasks. These tissues contain hepatocellular carcinoma with clear cell change from a pathologist’s perspective.

We also visualized the weight maps of TP53 mutation and p53 pathway of a representative patient in lung cancer in Fig. 5, and those of RB1 mutation and cell cycle pathway in the liver cancer in Fig. 6. In both examples, we can observe similar morphological patterns identified from a pathologist’s perspective in the two weight maps and tile appearances in the top 20 weighted tiles, which are similar to our observations for breast cancer. More results can be found in Supplementary Fig. 11 and Supplementary Fig. 12.

## Discussion

In this study, we demonstrated that key gene mutation outcomes and biological pathway activity of breast cancer can be predicted by deep-learning classifiers from whole-slide images. We further validated the deep-learning model to infer mutation status on liver and lung cancers, respectively. Our WSI-based deep-learning models can identify the point mutation status of six genes (RB1, CDH1, NF1, NOTCH2, TP53, and MAP3K1) and the copy number alteration of another six genes (FGFR1, EIF4EBP1, KAT6A, HEY1, ZNF217, and RAB25) in breast cancer. To deepen our understanding of cancer biology, we explored the predictive power of deep learning to predict underlying biological pathway activity, which is a challenging task involving complex biological relations among gene expressions. From the activity levels of 10 canonical signaling pathways derived from the mRNA expression data and copy number alteration inputs^6^, we found that three important pathways (p53, pi3k, and cell cycle) measured by mRNA expression and two pathways (Myc and Notch) measured by copy number alteration can be well predicted from our analysis.

Cancers are caused by gene mutations and therefore the prediction of key gene mutations based on whole-slide images will positively impact the targeted treatment of cancer patients^4,23–29^. For example, our models can predict TP53 point mutation (AUC 0.729) and FGFR1 copy number alteration (AUC 0.794) with high accuracies. TP53 is a tumor suppressor gene that plays a key role in many cellular pathways controlling cell proliferation, cell survival, and genomic integrity^23^. It is mutated frequently in breast cancer^4,23^ and has been associated with poor prognosis^4,23,24^. The FGFR1 gene is a member of the fibroblast growth factor receptor (FGFR) family that regulates important biological processes including cell proliferation and differentiation during development and tissue repair^25^. In breast cancer, FGFR1 amplification is the most frequent genomic aberration^26^, and may lead to dysregulated FGF receptors and promote cancer growth and metastasis. Extensive works^26–29^ have shown that FGFR1 could be a therapy target in breast cancer (e.g., the anti-FGFR1 dovitinib (TKI1258) therapy^27^). With the prediction of TP53 mutation and FGFR1 alteration, our models offered insights into selecting patient subgroups for the targeted therapy from digitalized WSI scans.

We extended our study to analyze biological pathway prediction based on whole-slide images that has seldom been addressed previously. Biological pathways are the interactions among molecules in a cell that result in certain products or changes in cancer^30^. Several important signaling pathways have been identified as frequently and genetically altered in cancer^18^. We showed that deep learning can predict pathology activity levels, providing valuable information for prognosis and therapeutic planning. For example, the p53 pathway activity (predicted with 0.798 AUC in our method) is associated with more aggressive disease and worse overall survival in breast cancer^31^. The Myc pathway (predicted with 0.795 AUC) acts as a key regulator of cell growth and proliferation, which has been linked to the basal-like breast cancer^32,33^, and can serve as a target for this aggressive subtype in breast cancer.

To overcome the interpretability challenges of AI-powered models, we employed a self-attention mechanism that is able to visualize the region of interest that contributed to outcomes prediction. In other words, we can display the weight map of each tumor tile to understand the decision making of the classifiers, highlighting the regions that contribute most to the final prediction. An example of the visualized weights map when predicting TP53 point mutation and p53 pathway activity is shown in Fig. 3b, c, respectively. Tiles with brighter green colors have larger weights, indicating that those tiles are most important in the decision-making process. Interestingly, the highlighted regions in both tasks are approximately located in the same part of the whole-slide image, and the top 20 weighted tiles in the two tasks shown in Fig. 3d, e, are also similar. The possible reason could be that TP53 is a crucial gene in the p53 pathway thus the predictions depend on similar image features in this example. This type of methodology and visualization has the potential to enable the improved exploration of the relationship between the image morphological features and molecular outcomes, and the relationship between genes and biological pathways, which can lead to new discoveries in breast cancer development.

To validate our method, we further extended the trained deep-learning models on lung and liver cancers with transfer learning. We hypothesize that the models trained using breast cancer data can also predict important gene mutations and pathway activities in lung and liver cancers since they are two common sites for the breast cancer metastatic spread^34^. Out of the well-predicted genes in breast cancer, the point mutations of TP53 and Notch2, and the copy number alteration of FGFR1 can also be predicted in lung cancer (LUAD). In the liver cancer (LIHC), the well-predicted genes are RB1 and TGF*β*2. These genes are indeed highly related to the diagnosis of lung cancer^19,35,36^ and liver cancer^20,37^. The different results on liver and lung cancers may be caused by the tissue differences of the two cancers. The pathway prediction results in the two cancers are not as good as gene mutation predictions, probably because pathways are more complicated than gene mutations thus are more challenging to predict. However, the well-predicted pathways in breast cancer still get the highest AUC scores in lung (p53) and liver (cell cycle, Myc) cohorts.

Overall, our study highlights deep characterization of breast cancer, its mutation outcome, and biological pathway activity. We present unique insights into WSI visual interactions between mutation and its pathway, enabling a head-to-head comparison to reinforce our major findings. Our approach can be a useful computational tool for gene mutation pre-screening, prior to the costly gene mutation analysis such as next-generation sequencing. Our evaluation strategy differs from pan-cancer studies^14–16^, which evaluate performance on each cancer individually. We measured the performance across cancer types by training on breast cancer and validating on liver and lung cancers, which is more challenging due to inherent differences of cancer tissues^38^. In terms of model development, we directly provide slide-level predictions without assuming that each tile or super-tile shares the same label as the whole slide. Unlike the regular attention mechanism used in the related works^39,40^ that calculates the weight of each patch according to the prediction, the self-attention in our work measures the similarities between each patch and all other patches and can capture the relationship between patches when making predictions. The self-attention mechanism further enables us to visualize the importance of tiles during the decision-making process, instead of the probabilities of mutations or expression for tiles. This finding can be used to better understand which image-based morphological features are related to certain gene mutations or pathway activities. Finally, our approach is a data-driven workflow that does not require nuclei detection^12^ as a prerequisite for specific prediction tasks.

While building associations between histopathology and molecular profiles is promising, the identified genotype–phenotype relationships here are not intended to replace standard transcriptomic tests. Given the confirmation from our collaborative pathologist that there is a lack of consensus on molecularly defined patterns seen from histopathological scans, we expect our detectable findings could complement pathologists’ routine workflow. The identified pathological descriptions were only exploratory rather than drawing conclusive associations, which warrant more clinical examinations in future studies. A limitation of the study is that the workflow is based on formalin-fixed, paraffin-embedded (FFPE) slides given their quality of preserving microscopic characteristics of tissues, while frozen tissues could also be considered for extended analysis in the future. Our computational analysis has a dependence on the feature extractor pretrained on natural images (ImageNet dataset^41^). There is a domain gap between natural images and pathology images. Therefore, the exploration of appropriate feature representations of pathology tiles and their parameters will be crucial to assess the validity and reproducibility of algorithms. To maximize the power of deep-learning approaches, it is also necessary to address data scarcity in histopathology-related tasks. There are often a significant portion of data samples that are insufficient and under-represented for certain mutation prediction tasks (e.g., only <5% mutant samples). High-quality, large-scale pathological data with precise molecular annotations will be needed to boost model development. Alternatively, transfer learning has proven to be useful in computer vision tasks when training samples are less available^42,43^. Therefore, a pretrained classifier built from diverse pathological datasets may provide superior results compared with our cancer-type-specific model.

In conclusion, we demonstrated that deep neural networks can be used to predict molecular outcomes in breast cancer including gene mutations and biological pathway activities from histopathology whole-slide images. Our extensive results highlighted new findings among genotype–phenotype associations, offering insights into the identification of targeted therapies for breast cancer treatment.

## Methods

### Data selection

The original TCGA-BRCA cohort consists of 1098 patients with H&E stained whole-slide images, genomic data, and additional clinical information. We analyzed the 1133 Formalin-Fixed Paraffin-Embedded (FFPE) slides that were generated by fixing a specimen in formaldehyde and then embedding it in a paraffin wax block for cutting. We further filtered out low quality FFPE slides according to the following criteria: (1) There is no diagnostic time information in a slide with low visual quality. (2) A slide has extensive blurred areas or is abnormally stained with little informative tissue areas. For patients with multiple slides, we only kept the slide with the best visual quality. After slide selection preprocessing, we collected 659 slides (659 patients) along with the corresponding omics data (mRNA expression and copy number alteration). The same selection process is performed on the validation datasets of TCGA-LUAD and TCGA-LIHC, resulting in 350 and 316 cases with both pathology images and omics profiles, respectively. The slide lists of the three cancers after data selection can be found in the Supplementary Note 1. These public TCGA cohorts were available online without restriction and authentication.

### Histopathology data preprocessing

**Fig. 7:**
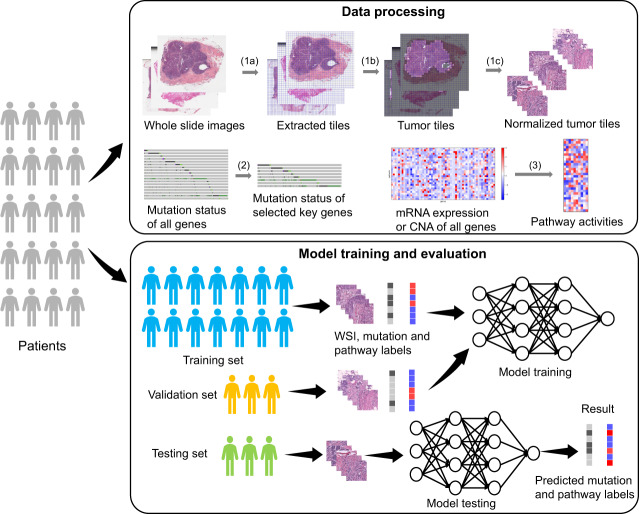
Illustration of the deep-learning workflow for data processing and model evaluation. We processed the WSI data by extracting tiles (1a), identifying tumor titles (1b), and generating small nonoverlapping tiles with color normalization. We selected key mutational genes (2) and identified biological pathways from mRNA (or CNA) expressions (3). Model training was based on a pretrained ResNet-101 model with an attention mechanism. After model selection, the trained model was used to test tiles and assess their prediction performances.

For each slide, we extracted nonoverlapping tiles of 512 × 512 at ×20 magnification and removed background tiles (Fig. 7a). A background tile was determined if its mean pixel value is higher than 220. We focused on tumor areas in the whole-slide images therefore we adopted a semi-automatic labeling method to identify tumor tiles. The labeling process was implemented by the initial clustering and manual refinement. In the first step, k-means clustering was performed on all tiles for each slide. Specifically, each 512 × 512 tile was downsampled to 128 × 128, which was then flattened into a 49152-length feature vector. These feature vectors were then clustered into two groups (i.e., tumor and nontumor regions). In the second step, a pathologist (20 years of clinical experience) additionally verified the segmentation quality of tumor regions and revised inaccurate results of slides to ensure the tumor labeling results were reasonable. For example, the tiles with artefacts in the annotated slides were removed manually if they were left after the clustering step. We then performed color normalization using the method^44^ to eliminate the color variations in different slides. The tiles of the same slide were processed by using the same slide-level pixel mean and standard deviation during the normalization.

### Mutated genes and pathway activity identification

To ensure a sufficient amount of training WSIs for mutated genes, for point mutation we selected 18 important genes in breast cancer, which were related to cell functions (e.g., cell cycle, p53 signaling, notch signaling, and DNA damage response) and they were mutated at least 3%. For CNA data, we selected 35 genes with mutation percentage greater than 5%. In the validation tasks of lung and liver cancers, we used the same criterion to select gene profiles, resulting in 9 point mutation genes and 14 CNA genes in the lung cancer and 7 point mutation genes and 25 CNA genes in the liver cancer for analysis.

In the pathway activity prediction task, we identified ten canonical signaling pathways with frequent genetic alterations. The pathway activity in each patient was obtained by a weighted sum of the genes’ expression data or CNA data in the pathway. Then the activity was binarized as activated if it is greater than zero and inactivated otherwise. For each pathway, we generated two types of activity labels from mRNA expression data or CNA data for each patient as follow:

1 $$\begin{array}{*{20}{c}} {v^{s,i} = \frac{1}{{N_{gene}^i}}\mathop {\sum }\limits_{n = 1}^{N_{gene}^i} w_n^{s,i}u_n^{s,i},i = 1,2, \ldots ,10,s = 1,2, \ldots ,659} \end{array}$$

2 $$\begin{array}{*{20}{c}} {l^{s,i} = \left\{ {\begin{array}{*{20}{c}} {1\;if\;v^{s,i}\, >\, 0} \\ {0\;{\mathrm{otherwise}}} \end{array}} \right.,} \end{array}$$

where *v*^*s*,*i*^ is the activity level of pathway *i* in patient *s*, *l*^*s,i*^ is the binary activity label of pathway *i* in patient *s*, $N_{{\mathrm{gene}}}^i$ is the number of important genes that are involved in the pathway *i* according to Sanchez-Vega et al.’s work^18^, $u_n^{s,i}$ is the expression level or CNA level of gene *n* in pathway *i* and patient *s*, $w_n^{s,i}$ is the corresponding weight, which takes value 1 if the gene is an oncogene and −1 if it is a tumor suppressor. The CNN aims to predict the binary label *l*^*s,i*^, i.e., whether a pathway is activated (*l*^*s,i*^ = 1) or inactivated (*l*^*s,i*^ = 0) in a patient.

### Model structure

We developed deep-learning models to predict mutation status and pathway activity from histopathology images. Our model architecture consists of two main sections (Fig. 1):

- Feature extractor: This module aims to obtain a feature vector representing the input tile. We use the convolutional layers of ResNet-101^45^ as the feature extractor, which is widely used in image classification tasks and has shown powerful feature representation ability in various applications. This subnetwork is pretrained on the ImageNet dataset^41^ and kept unchanged during training and testing. Through the feature extraction, each input tile is represented by a 2048-dimensional feature vector, resulting in a feature matrix of *N* × 2048 for slide of patient *s*, where *N* is the number of tumor tiles in the slide and varies from slide to slide.
- Multi-layer perceptron (MLP) predictor with self-attention: This subnetwork follows the feature extractor to output the final prediction. It consists of three fully connected layers and one self-attention layer (Fig. 1). The first two fully connected layers have 512 and 128 neurons, respectively, reducing the size of feature matrix to *N* × 128. The self-attention layer is used to compute the importance weight of each tile’s feature vector and guide the network to pay more attention to the crucial tiles. Self-attention has been used successfully in a variety of tasks in natural language processing^46–48^ and computer vision^49^ to model relationships between widely separated spatial regions. In this paper, we make slight modifications based on the method in Zhang et al.^49^:

3 $$\begin{array}{*{20}{c}} {f\left( x \right) = W_fx,g\left( x \right) = W_gx} \end{array}$$

4 $$\begin{array}{*{20}{c}} {\alpha _{i,j} = {\mathrm{softmax}}\left( {f\left( {x_i} \right)^Tg\left( {x_j} \right)} \right)} \end{array}$$

5 $$\begin{array}{*{20}{c}} {o_j = \mathop {\sum }\limits_{i = 1}^N \alpha _{j,i}x_i,y = x + \gamma \cdot o} \end{array}$$

where *x* is the input feature matrix, *W*_*f*_ and *W*_*g*_ are 1 × 1 convolution filters, *α*_*j,i*_ indicates how much attention the model pays to the *i*th tile’s features when computing the *j*th tile’s activation *o*_*j*_, *γ* is a trainable parameter controlling the scale of the attention. *y* is the output of the self-attention layer after an average pooling, which is the global feature vector representing all tumor tiles of a slide. The final fully connected layer transforms the global feature to a prediction.

In our study, the gene mutation status prediction and pathway activity prediction are formulated as classification tasks, and thus cross-entropy loss is used to train the models.

### Model training and evaluation

The feature extractor subnetwork (ResNet-101 without fully connected (FC) layer) is pretrained and fixed during training for all prediction tasks. Therefore, we extract the feature vectors of tumor tiles for all patients beforehand and save them to the disk. Training the prediction module from the saved feature vectors can greatly accelerate the training speed. During feature extraction, each 512 × 512 tile is resized to 224 × 224 image and normalized by the mean and standard deviation of the ImageNet dataset^39^ before feeding to the pretrained ResNet-101. The prediction subnetwork is trained with the Adam optimizer for 30 epochs. The initial value of *γ* in the self-attention layer is 1. The learning rate of *γ* is set to 0.001 and all other parameters have a learning rate of 0.0001. The best model is saved when achieving the best performance on the validation set. For different tasks (e.g., point mutation, pathway activity), the models for breast cancer are all trained from scratch. It took ~6 min to train the MLP with self-attention (30 epochs, batch size 8) on a NVIDIA TITAN Xp GPU. Our training is efficient because our method can directly provide slide-level prediction instead of tile-level predictions as done in Fu et al^15^.

To evaluate the model’s performance on the validation set (for model selection) and test set, we use the area-under-the-curve (AUC) in both mutation prediction and pathway activity prediction tasks. The AUC is the area under the ROC curve, which is created by plotting the true positive rate (TPR) against the false positive rate (FPR) at various threshold settings. AUC informs the capability of a model in distinguishing between classes. The 95% confidence interval (CI) of each AUC score is calculated by 1000 bootstrapping to estimate the uncertainty of AUC.

### Model fine-tuning on the lung and liver cancers data

During model fine-tuning, we fix the parameters of the first two fully connected (fc) layers of the prediction subnetwork and fine-tune the self-attention layer and the last fc layer. We assume that image features learned from breast cancer data could be also useful in a pan-cancer setting. This fine-tuning strategy could help to investigate if there is any underlying relationship between the data of breast cancer and lung or liver cancer. We did not fine-tune models for all possible gene profiles from TCGA, because the point mutation and CNA percentage of some genes are extremely low in lung or liver cancers. Only genes with >3% point mutation or >5% CNA were fine-tuned and tested in our study.

### Visualization

The self-attention layer in our model can produce the importance weights of tiles in a slide in the prediction tasks, which is helpful for us to explore the biological interpretation value of deep-learning classifiers. We compute the log value of the weight of each tile and project it to the original location in the whole-slide image, resulting in the weight map (Fig. 3). The weight *β*_*i*_ is computed according to Eqs. (3), (4), (5):

6 $$\begin{array}{*{20}{c}} {\beta _i = 1 + \gamma \mathop {\sum }\limits_{j = 1}^N \alpha _{i,j}} \end{array}$$

Besides, we select tiles with top 20 largest weights in a slide to show the appearance of important tiles (Figs. 3d and e).

### Reporting summary

Further information on research design is available in the Nature Research Reporting Summary linked to this article.

## Supplementary information

Supplementary Information

Reporting Summary

## Data Availability

The whole-slide images used in this study are publicly available through the Genomic Data Commons data portal (https://portal.gdc.cancer.gov/). The omics data (mutation, copy number alteration and mRNA expression data) are publicly available through cBioPortal (https://www.cbioportal.org/), and the download links are provided in the Supplementary Note 2.

## Code availability

The source code used in this study can be found at https://github.com/huiqu18/GeneMutationFromHE.
