# Supplementary material for: Genetic mutation and biological pathway prediction based on whole slide images in breast carcinoma using deep learning
Source: NPJ Precis Oncol. 2021 Sep 23;5:87. doi: 10.1038/s41698-021-00225-9 (PMC8460699; doi:10.1038/s41698-021-00225-9)
Supplement: Supplementary file 1 — Supplementary Information [file 41698_2021_225_MOESM1_ESM.pdf]

Genetic Mutation and Biological Pathway Prediction based on Whole Slide Images in Breast Carcinoma using Deep Learning

Hui Qu[1], Mu Zhou[2], Zhennan Yan[2], He Wang[3], Vinod K. Rustgi[4], Shaoting Zhang[5], Olivier Gevaert[6], Dimitris N. Metaxas[1]

Supplementary Figures

A. Five more examples of weight maps when predicting the point mutation status of **TP53 and p53** pathway activity from mRNA expression data in **breast cancer.**

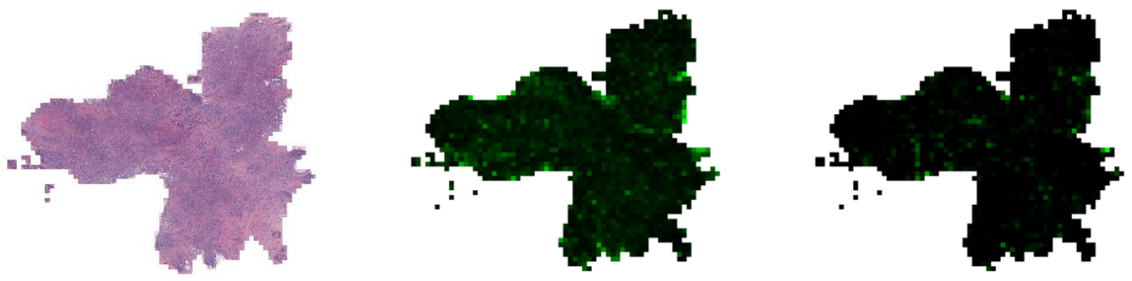

(a) tumor tiles (b) TP53 mutation (c) p53 pathway activity

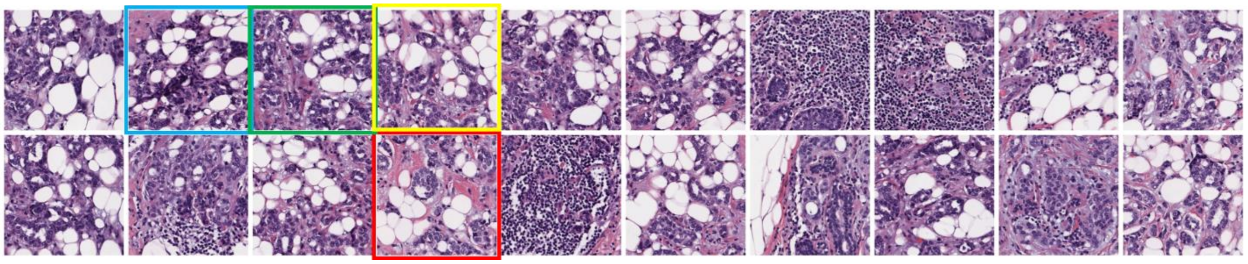

(d) Top 20 weighted tiles in TP53 mutation prediction

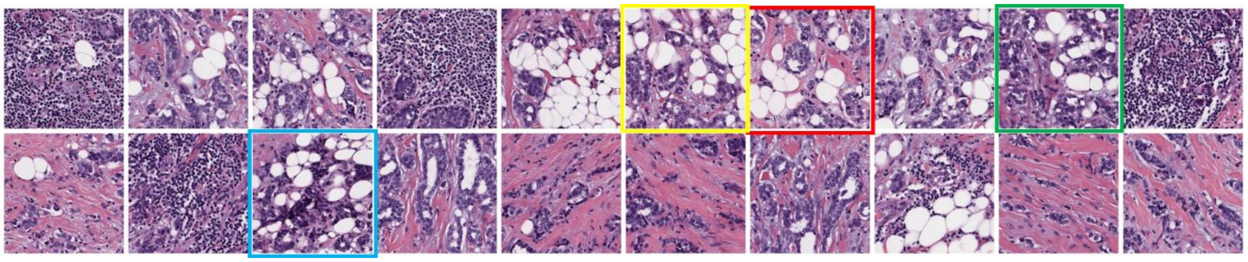

(e) Top 20 weighted tiles in p53 pathway activity prediction

Supplementary Figure 1. Weight maps of tiles when predicting the point mutation status of TP53 and p53 pathway activity from mRNA expression data in breast cancer. (a) Tumor tiles after data processing. (b) Weight map of tumor tiles in TP53 point mutation prediction. Brighter green tiles have larger weights. (c) Weight map of p53 pathway activity prediction. (d) and (e): Top 20 weighted tiles for TP53 point mutation prediction and p53 pathway prediction, respectively. We marked four tiles that appear in both tasks.

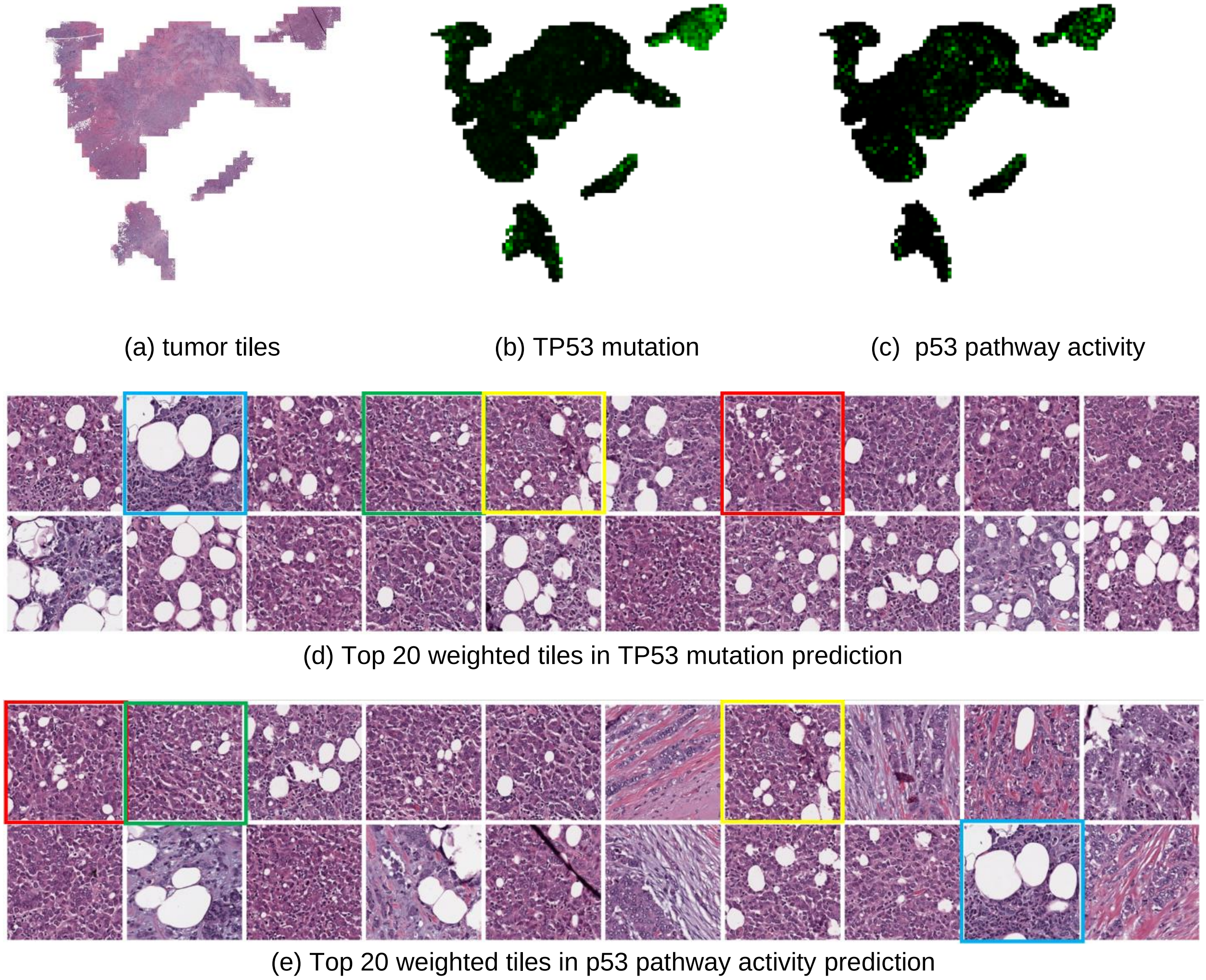

Supplementary Figure 2. Weight maps of tiles when predicting the point mutation status of TP53 and p53 pathway activity from mRNA expression data in breast cancer. (a) Tumor tiles after data processing. (b) Weight map of tumor tiles in TP53 point mutation prediction. Brighter green tiles have larger weights. (c) Weight map of p53 pathway activity prediction. (d) and (e): Top 20 weighted tiles for TP53 point mutation prediction and p53 pathway prediction, respectively. We marked four tiles that appear in both tasks.

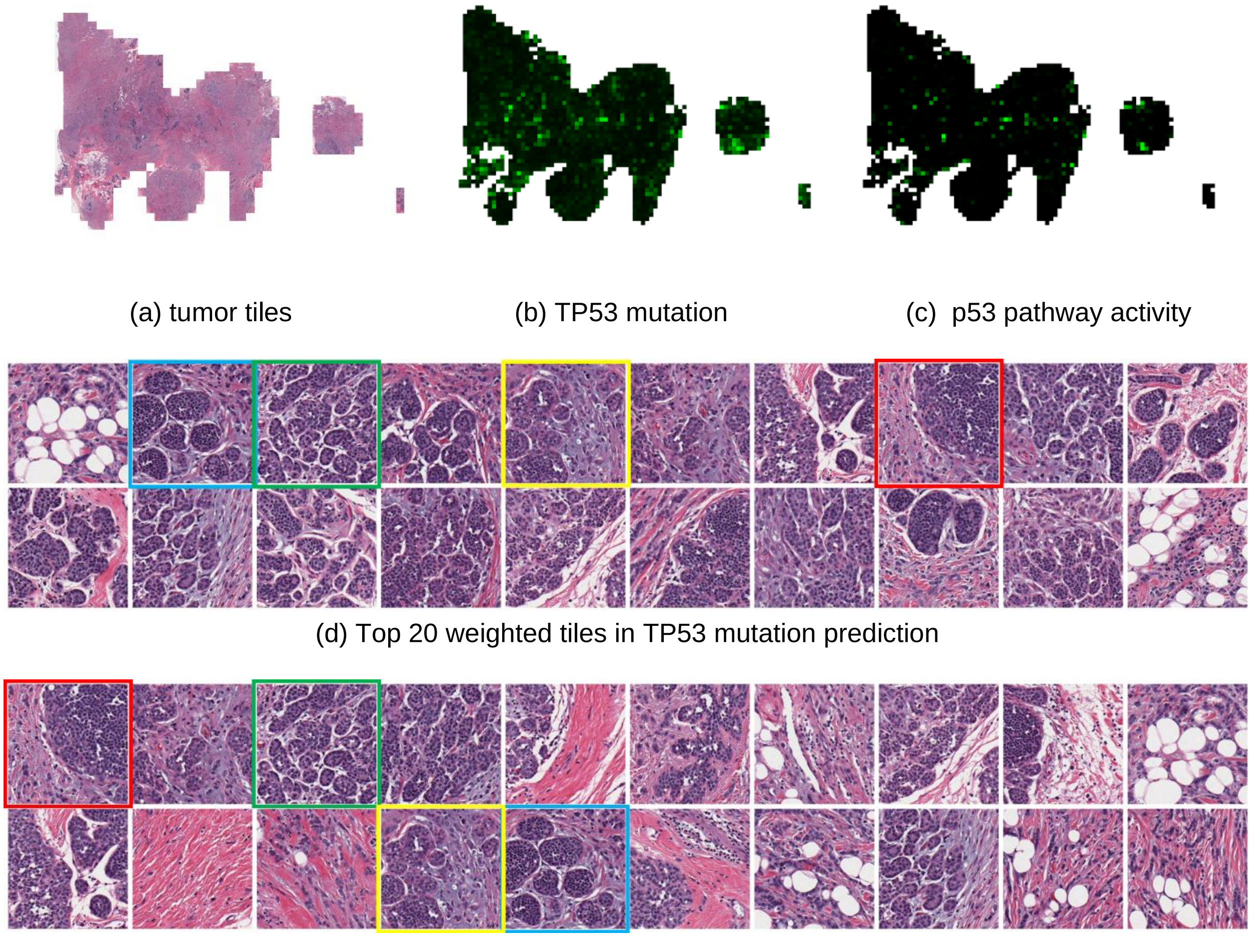

(a) tumor tiles (b) TP53 mutation (c) p53 pathway activity

(d) Top 20 weighted tiles in TP53 mutation prediction

(e) Top 20 weighted tiles in p53 pathway activity prediction

Supplementary Figure 3. Weight maps of tiles when predicting the point mutation status of TP53 and p53 pathway activity from mRNA expression data in breast cancer. (a) Tumor tiles after data processing. (b) Weight map of tumor tiles in TP53 point mutation prediction. Brighter green tiles have larger weights. (c) Weight map of p53 pathway activity prediction. (d) and (e): Top 20 weighted tiles for TP53 point mutation prediction and p53 pathway prediction, respectively. We marked four tiles that appear in both tasks.

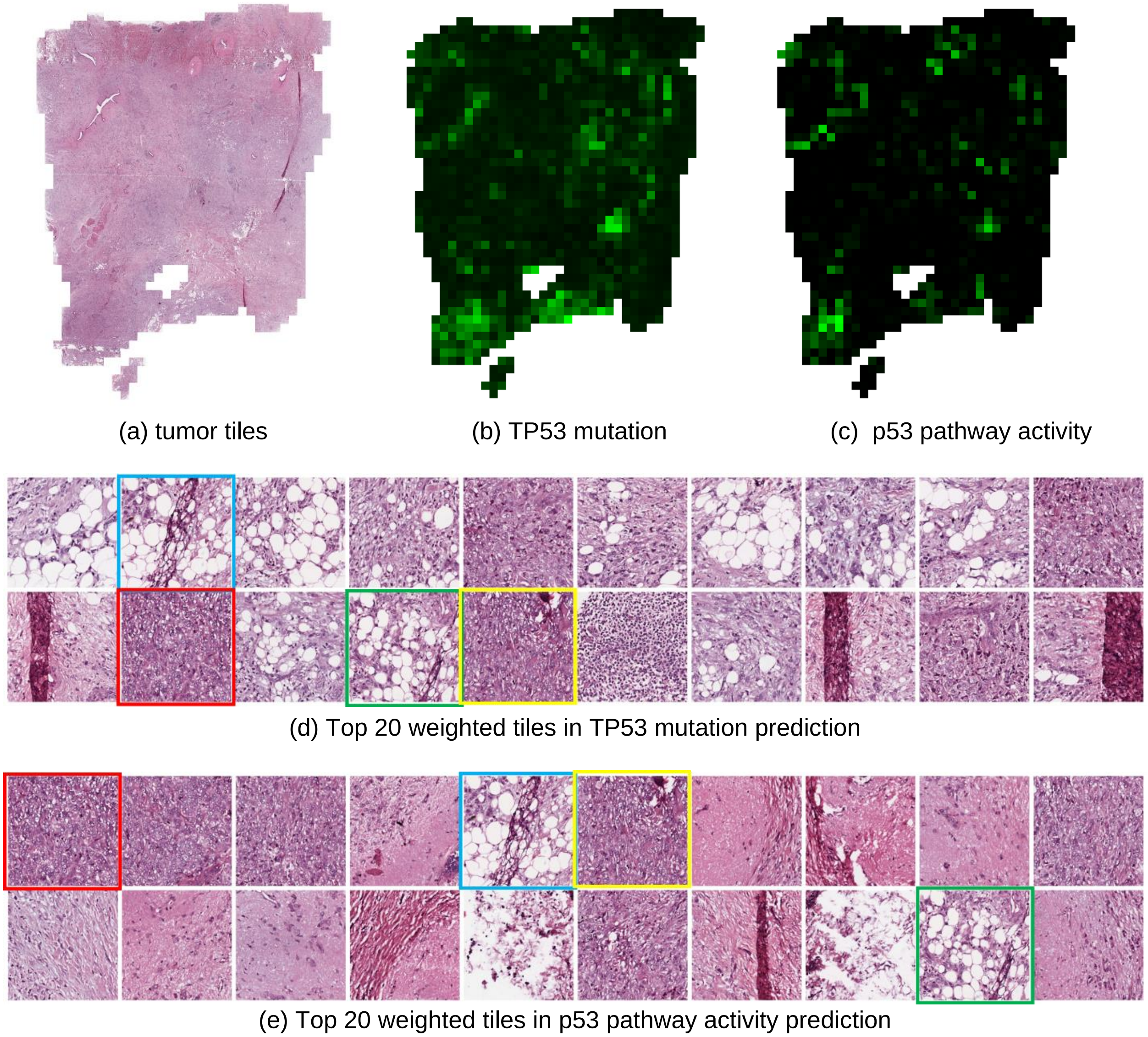

Supplementary Figure 4. Weight maps of tiles when predicting the point mutation status of TP53 and p53 pathway activity from mRNA expression data in breast cancer. (a) Tumor tiles after data processing. (b) Weight map of tumor tiles in TP53 point mutation prediction. Brighter green tiles have larger weights. (c) Weight map of p53 pathway activity prediction. (d) and (e): Top 20 weighted tiles for TP53 point mutation prediction and p53 pathway prediction, respectively. We marked four tiles that appear in both tasks.

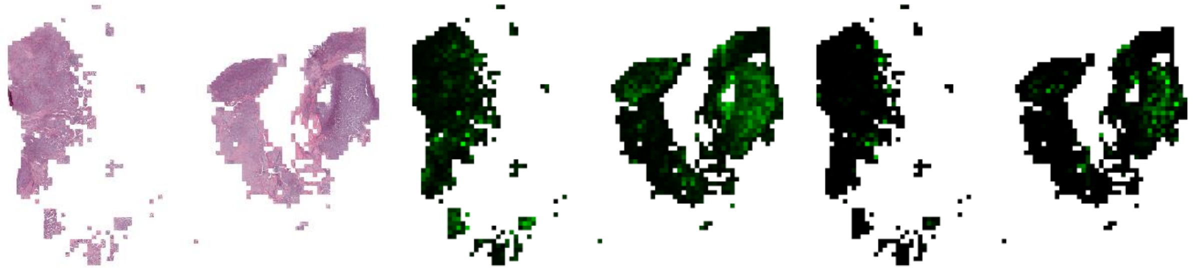

(a) tumor tiles (b) TP53 mutation (c) p53 pathway activity

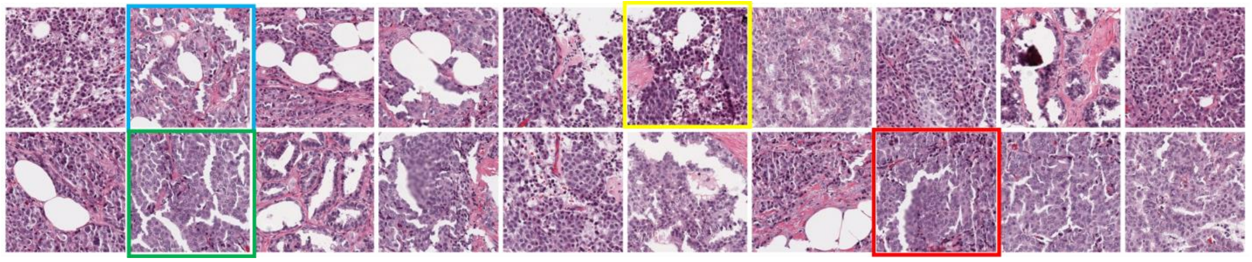

(d) Top 20 weighted tiles in TP53 mutation prediction

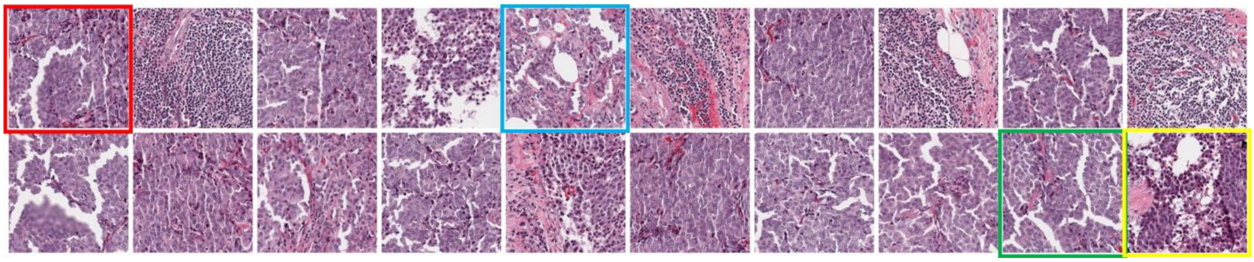

(e) Top 20 weighted tiles in p53 pathway activity prediction

Supplementary Figure 5. Weight maps of tiles when predicting the point mutation status of TP53 and p53 pathway activity from mRNA expression data in breast cancer. (a) Tumor tiles after data processing. (b) Weight map of tumor tiles in TP53 point mutation prediction. Brighter green tiles have larger weights. (c) Weight map of p53 pathway activity prediction. (d) and (e): Top 20 weighted tiles for TP53 point mutation prediction and p53 pathway prediction, respectively. We marked four tiles that appear in both tasks.

B. Five more examples of weight maps when predicting the point mutation status of **RB1 and cellcycle** pathway activity from mRNA expression data in **breast cancer.**

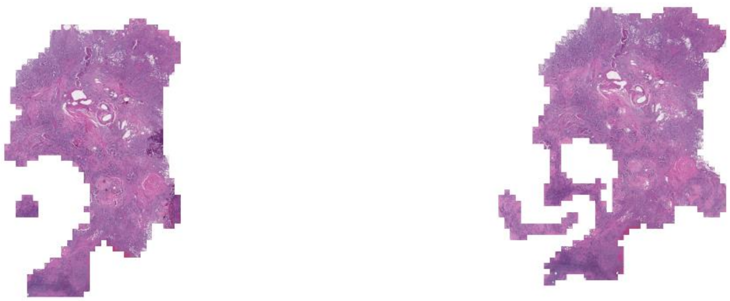

(a) tumor tiles

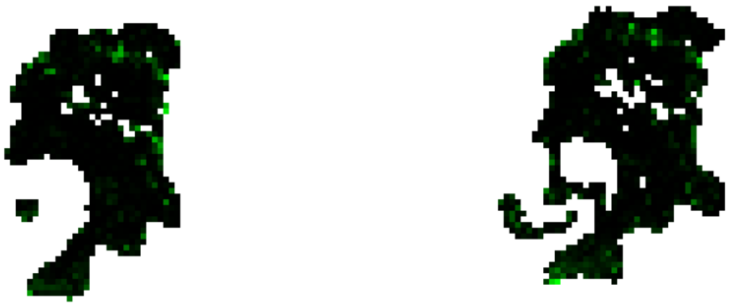

(b) RB1 mutation

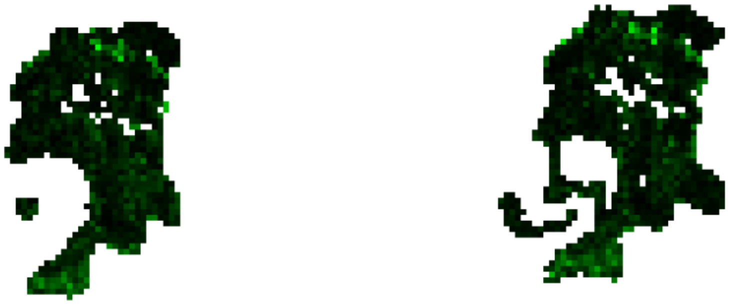

(c) cell cycle pathway activity

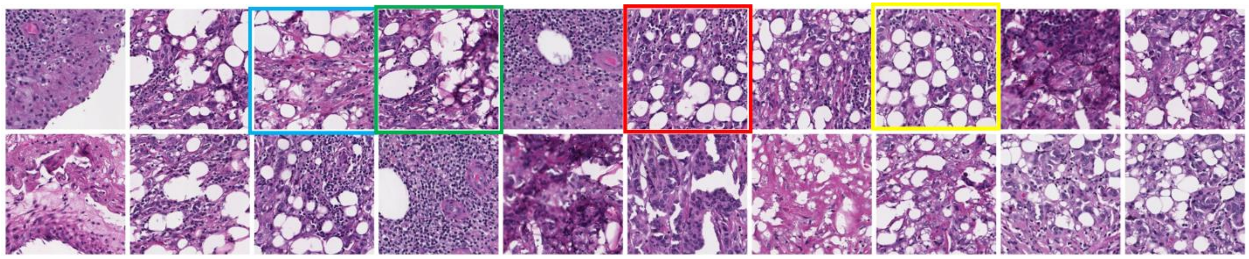

(d) Top 20 weighted tiles in RB1 mutation prediction

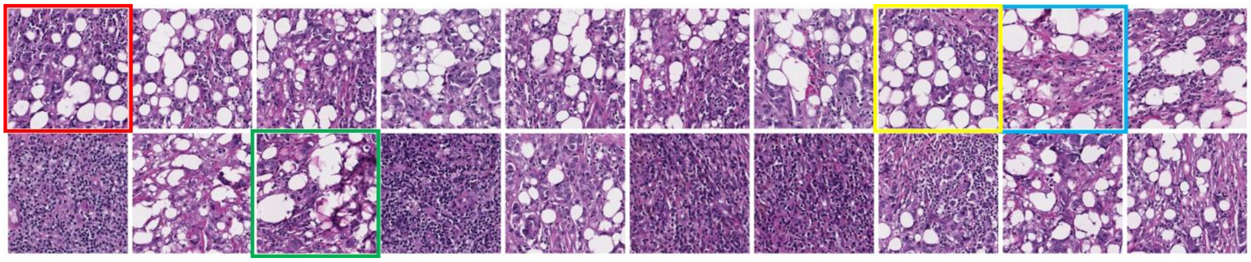

(e) Top 20 weighted tiles in cell cycle pathway activity prediction

Supplementary Figure 6. Weight maps of tiles when predicting the point mutation status of RB1 and cell cycle pathway activity from mRNA expression data in breast cancer. (a) Tumor tiles after data processing. (b) Weight map of tumor tiles in RB1 point mutation prediction. Brighter green tiles have larger weights. (c) Weight map of the cell cycle pathway activity prediction. (d) and (e): Top 20 weighted tiles for the RB1 point mutation prediction and cell cycle pathway prediction, respectively. We marked four tiles that appear in both tasks.

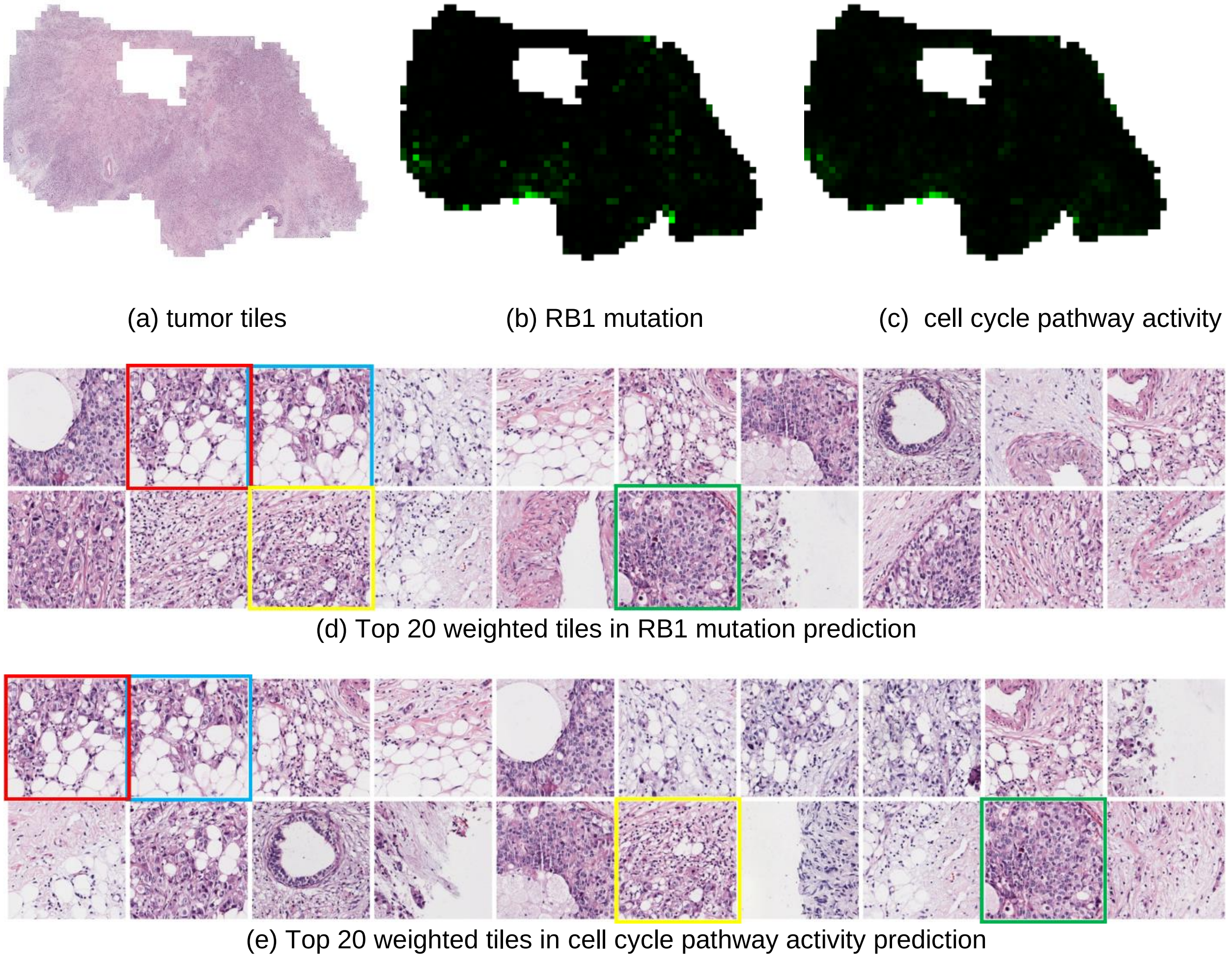

Supplementary Figure 7. Weight maps of tiles when predicting the point mutation status of RB1 and cell cycle pathway activity from mRNA expression data in breast cancer. (a) Tumor tiles after data processing. (b) Weight map of tumor tiles in RB1 point mutation prediction. Brighter green tiles have larger weights. (c) Weight map of the cell cycle pathway activity prediction. (d) and (e): Top 20 weighted tiles for the RB1 point mutation prediction and cell cycle pathway prediction, respectively. We marked four tiles that appear in both tasks

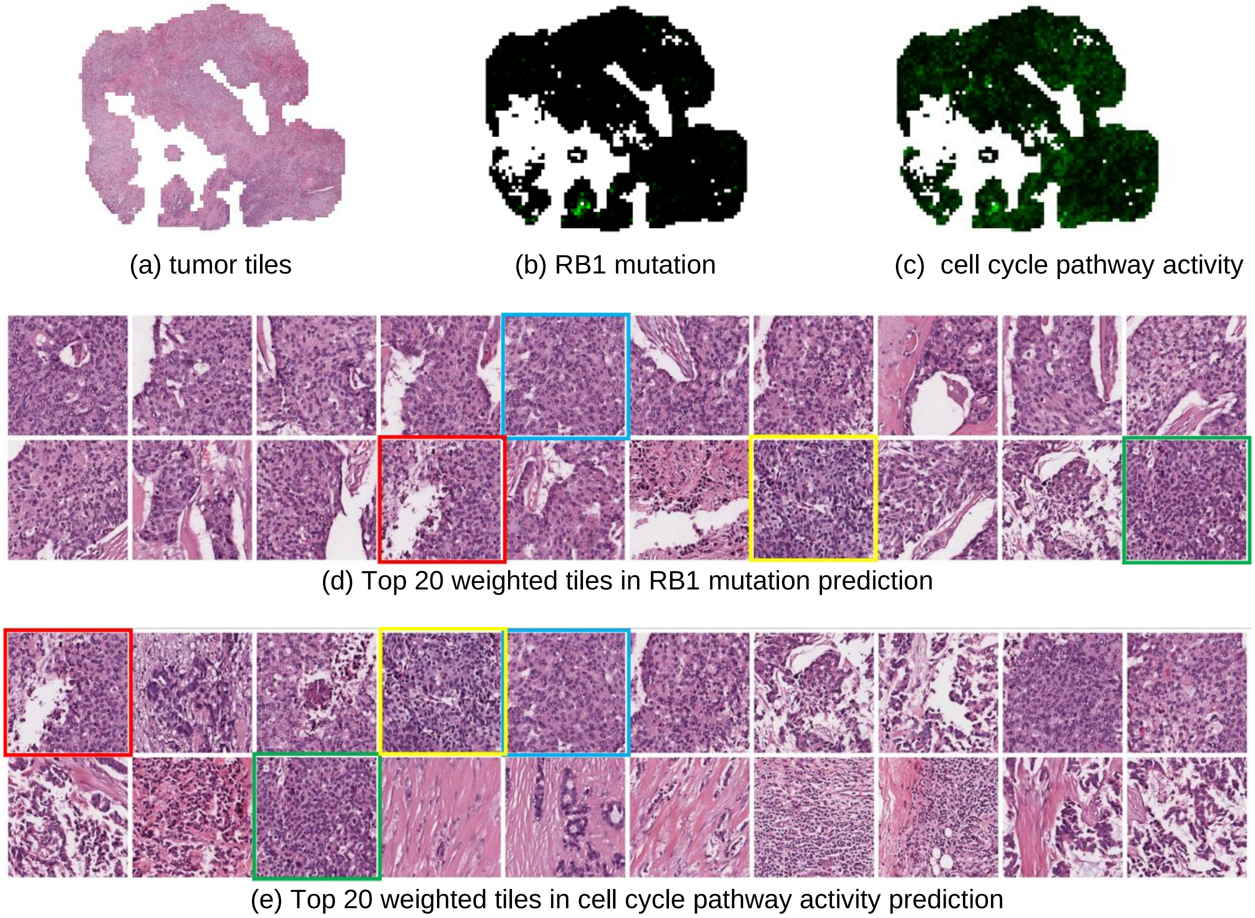

Supplementary Figure 8. Weight maps of tiles when predicting the point mutation status of RB1 and cell cycle pathway activity from mRNA expression data in breast cancer. (a) Tumor tiles after data processing. (b) Weight map of tumor tiles in RB1 point mutation prediction. Brighter green tiles have larger weights. (c) Weight map of the cell cycle pathway activity prediction. (d) and (e): Top 20 weighted tiles for the RB1 point mutation prediction and cell cycle pathway prediction, respectively. We marked four tiles that appear in both tasks

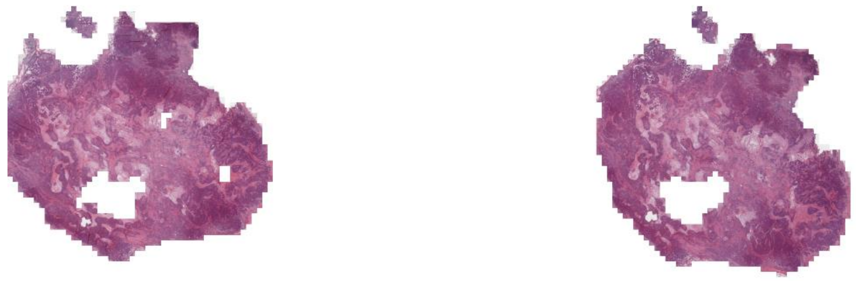

(a) tumor tiles

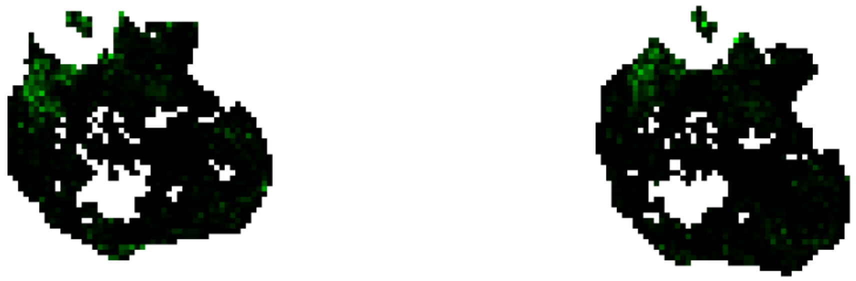

(b) RB1 mutation

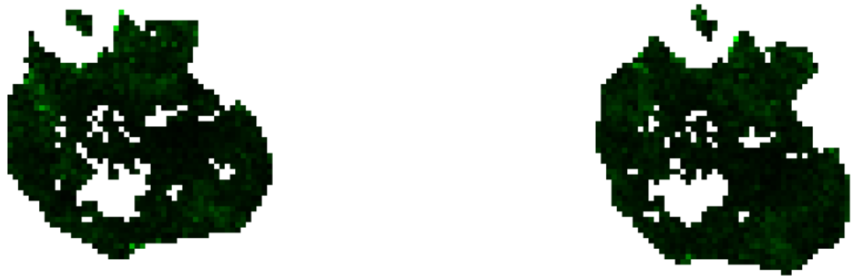

(c) cell cycle pathway activity

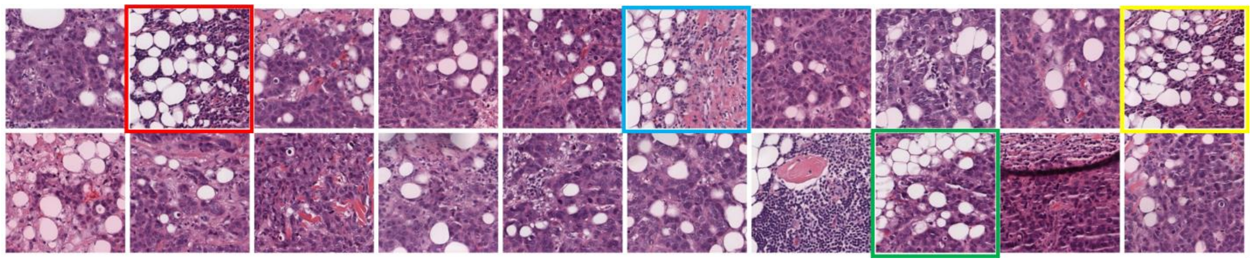

(d) Top 20 weighted tiles in RB1 mutation prediction

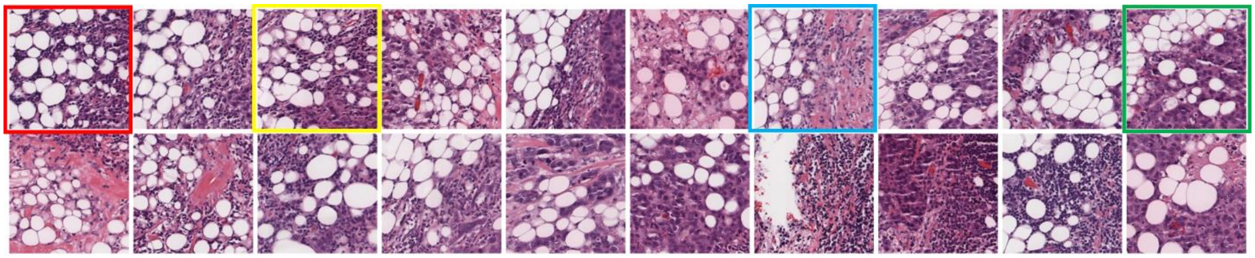

(e) Top 20 weighted tiles in cell cycle pathway activity prediction

Supplementary Figure 9. Weight maps of tiles when predicting the point mutation status of RB1 and cell cycle pathway activity from mRNA expression data in breast cancer. (a) Tumor tiles after data processing. (b) Weight map of tumor tiles in RB1 point mutation prediction. Brighter green tiles have larger weights. (c) Weight map of the cell cycle pathway activity prediction. (d) and (e): Top 20 weighted tiles for the RB1 point mutation prediction and cell cycle pathway prediction, respectively. We marked four tiles that appear in both tasks

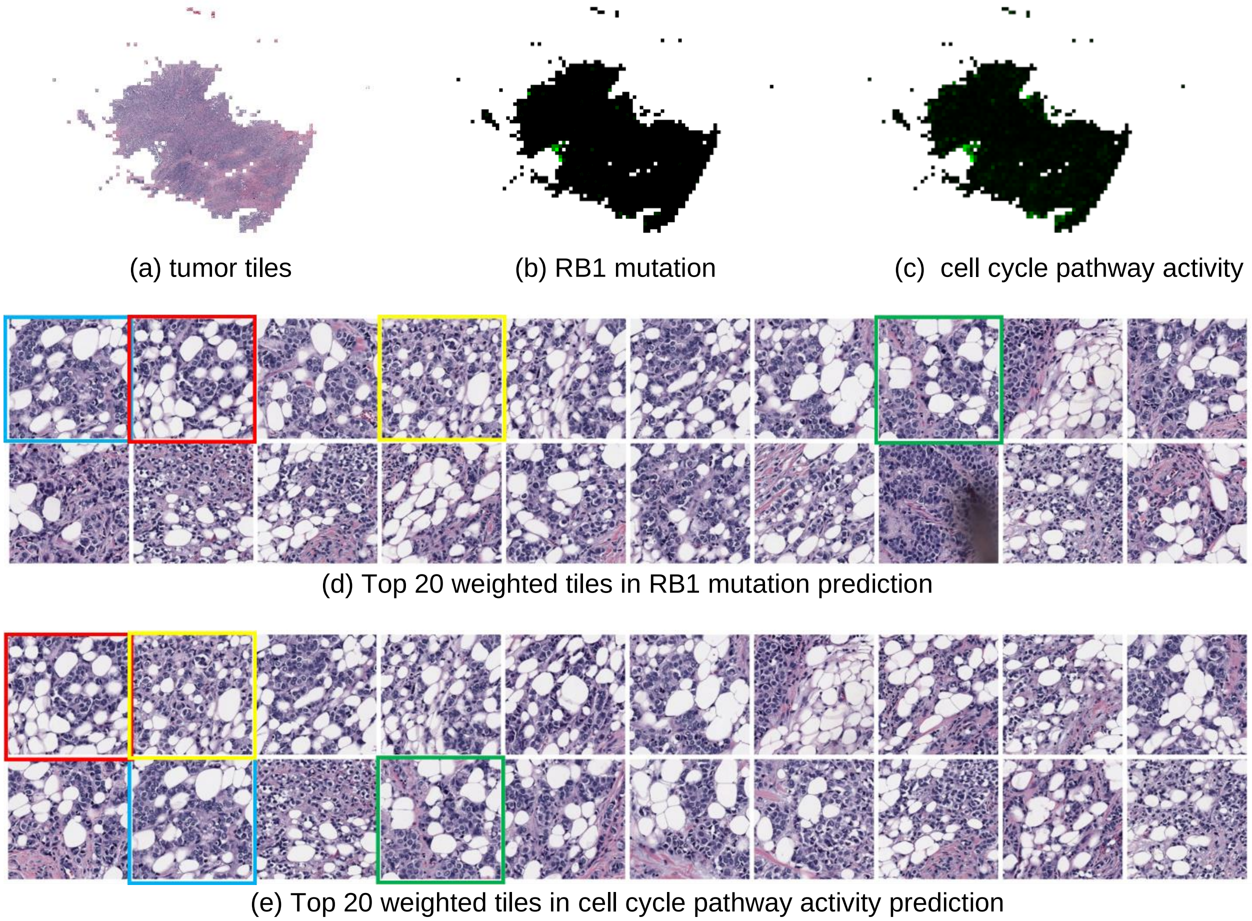

Supplementary Figure 10. Weight maps of tiles when predicting the point mutation status of RB1 and cell cycle pathway activity from mRNA expression data in breast cancer. (a) Tumor tiles after data processing. (b) Weight map of tumor tiles in RB1 point mutation prediction. Brighter green tiles have larger weights. (c) Weight map of the cell cycle pathway activity prediction. (d) and (e): Top 20 weighted tiles for the RB1 point mutation prediction and cell cycle pathway prediction, respectively. We marked four tiles that appear in both tasks

C. One more example of weight maps when predicting the point mutation status of **TP53 and p53** pathway activity from mRNA expression data in **lung cancer.**

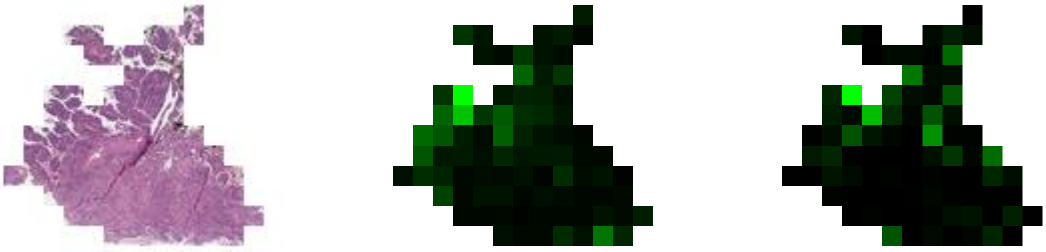

(a) tumor tiles (b) TP53 mutation (c) p53 pathway activity

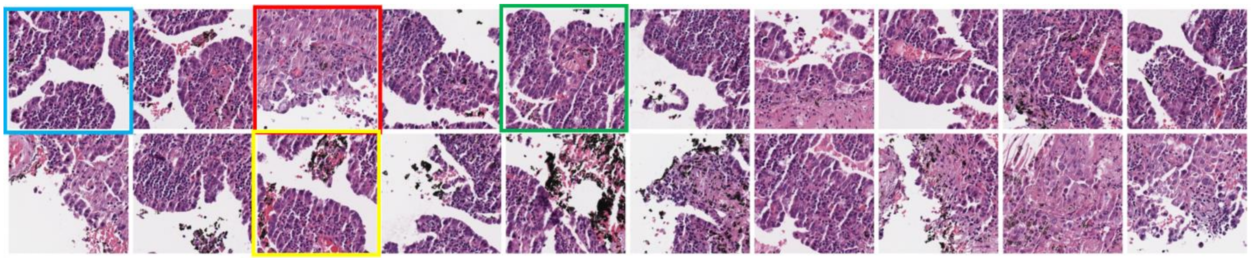

(d) Top 20 weighted tiles in TP53 mutation prediction

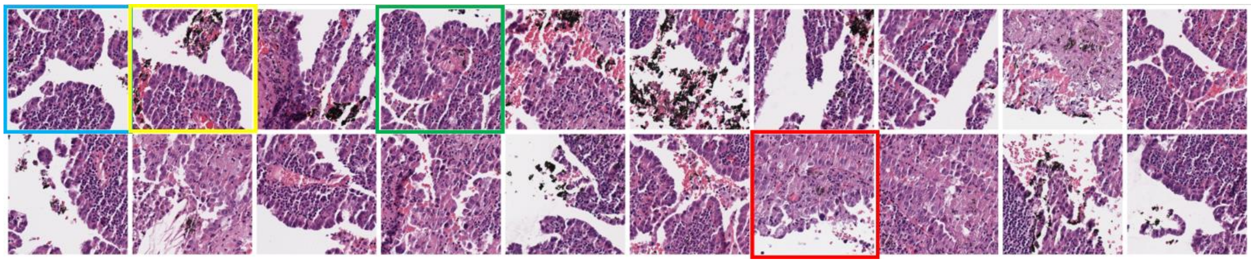

(e) Top 20 weighted tiles in p53 pathway activity prediction

Supplementary Figure 11. Weight maps of tiles when predicting the point mutation status of TP53 and p53 pathway activity from mRNA expression data in lung cancer. (a) Tumor tiles after data processing. (b) Weight map of tumor tiles in TP53 point mutation prediction. Brighter green tiles have larger weights. (c) Weight map in p53 pathway activity prediction. (d) and (e): Top 20 weighted tiles for the TP53 point mutation prediction and p53 pathway prediction, respectively. We marked four tiles that appeared in both tasks.

D. One more examples of weight maps when predicting the point mutation status of **RB1 and cellcycle** pathway activity from mRNA expression data in **liver cancer.**

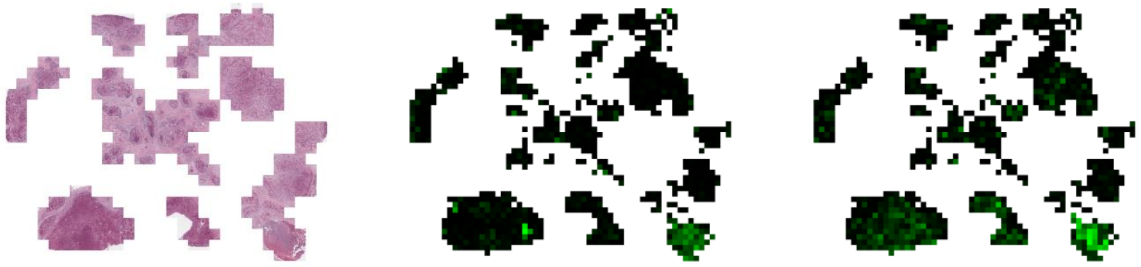

(a) tumor tiles (b) RB1 mutation (c) cell cycle pathway activity

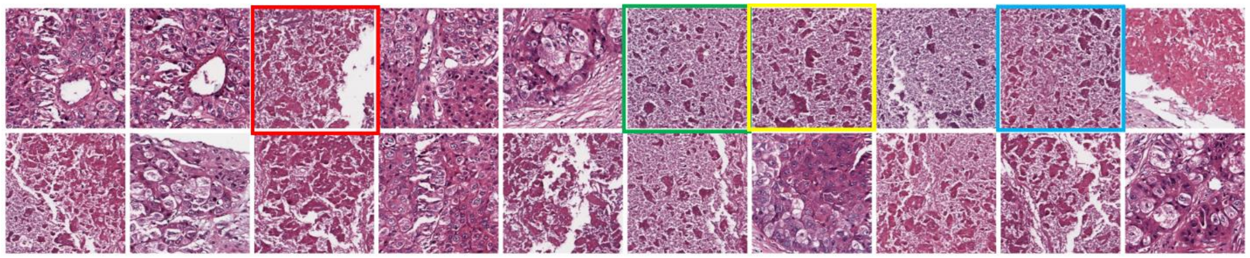

(d) Top 20 weighted tiles in RB1 mutation prediction

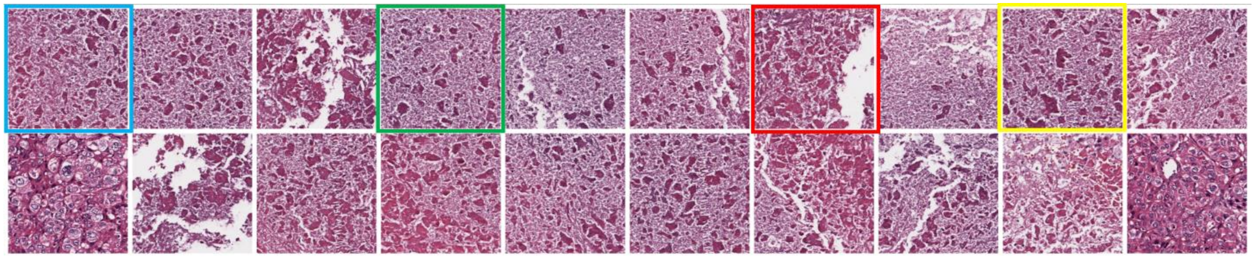

(e) Top 20 weighted tiles in cell cycle pathway activity prediction

Supplementary Figure 12. Weight maps of tiles when predicting the point mutation status of RB1 and cell cycle pathway activity from mRNA expression data in liver cancer. (a) Tumor tiles after data processing. (b) Weight map of tumor tiles in RB1 point mutation prediction. Brighter green tiles have larger weights. (c) Weight map in cell cycle pathway activity prediction. (d) and (e): Top 20 weighted tiles from RB1 point mutation prediction and cell cycle pathway prediction, respectively. We marked four tiles that appeared in both tasks.

Supplementary Tables

Supplementary Table 1. AUC (with 95% CI) of the models trained on the point mutation data of breast cancer, and finetuned on the lung and liver cancers. Genes with empty results in LUAD and LIHC contain too few mutated samples, thus we didn't predict their mutations.

Gene	BRCA		LUAD		LIHC	
	Mutation percentage	Slide AUC (95% CIs)	Mutation percentage	Slide AUC (95% CIs)	Mutation percentage	Slide AUC (95% CIs)
ARID1A	4.40 %	42.71 (12.24, 73.49)	6.00 %	48.66 (34.85, 62.62)	8.23 %	54.39 (43.31, 65.77)
CDH1	9.86 %	77.57 (62.51, 91.35)	0.86 %	-	1.58 %	-
CTCF	3.03 %	30.58 (0.00, 52.02)	1.43 %	-	0.95 %	-
ERBB2	3.64 %	62.47 (31.90, 82.48)	1.14 %	-	0.32 %	-
GATA3	12.29 %	63.76 (46.79, 80.18)	2.29 %	-	0.32 %	-
KMT2C	7.74 %	42.93 (11.34, 81.96)	13.14 %	46.09 (36.51, 55.10)	5.06 %	44.06 (29.28, 58.68)
MAP2K4	4.70 %	55.15 (20.41, 81.45)	1.14 %	-	1.58 %	-
MAP3K1	7.28 %	68.17 (41.93, 94.85)	0.86 %	-	0.63 %	-
NF1	3.64 %	76.78 (44.90, 94.85)	14.00 %	55.85 (47.02, 64.29)	3.80 %	65.09 (40.01, 86.51)
NOTCH2	3.03 %	74.01 (51.48, 91.67)	4.29 %	65.60 (52.80, 78.47)	1.90 %	-
PIK3CA	30.80 %	58.00 (44.63, 72.44)	5.43 %	45.90 (30.35, 63.03)	2.53 %	-
PTEN	4.70 %	52.74 (15.46, 92.86)	1.71 %	-	1.27 %	-
RB1	3.03 %	85.16 (73.95, 96.94)	6.00 %	52.55 (37.22, 68.20)	5.06 %	79.49 (67.45, 89.34)
RUNX1	4.40 %	33.04 (9.57, 58.77)	0.57 %	-	0.00 %	-
RYR2	6.22 %	47.25 (13.40, 91.78)	38.29 %	57.69 (50.56, 64.47)	9.81 %	57.79 (44.15, 69.83)
TBX3	3.19 %	45.12 (14.29, 94.85)	2.00 %	-	1.27 %	-
TP53	35.96 %	72.88 (62.05, 82.78)	52.57 %	70.50 (64.25, 76.25)	30.38 %	55.46 (48.23, 62.50)
USH2A	5.46 %	49.78 (30.89, 69.01)	31.71 %	50.34 (43.07, 58.05)	6.96 %	45.97 (29.93, 62.82)

Supplementary Table 2a. AUC (with 95% CI) of the models trained on the copy number alteration data of breast cancer, and finetuned on the lung and liver cancers (Part a). Genes with empty results in LUAD and LIHC contain too few mutated samples, thus we didn't predict their copy number alteration.

Gene	BRCA		LUAD		LIHC	
	Mutation percentage	Slide AUC (95% CIs)	Mutation percentage	Slide AUC (95% CIs)	Mutation percentage	Slide AUC (95% CIs)
AKT3	9.7%	45.02 (24.25, 67.72)	4.57 %	-	7.59 %	63.04 (49.40, 77.00)
APH1A	10.6%	59.61 (41.58, 75.27)	11.14 %	43.90 (34.04, 53.75)	10.76 %	52.58 (37.71, 65.62)
BMP7	5.6%	48.01 (18.55, 89.80)	3.43 %	-	1.27 %	-
CCND1	16.2%	37.64 (21.56, 55.08)	2.86 %	-	6.96 %	60.70 (48.93, 71.85)
CDKN2A	5.2%	49.14 (27.58, 70.21)	15.43 %	43.51 (34.78, 53.08)	5.70 %	57.87 (41.11, 71.73)
CDKN2B	5.0%	43.64 (31.85, 57.02)	14.86 %	48.09 (39.12, 56.91)	5.06 %	60.05 (41.38, 76.02)
E2F5	8.3%	45.24 (22.22, 68.35)	3.43 %	-	7.91 %	47.05 (35.96, 59.11)
EIF4EBP1	14.1%	74.20 (59.50, 87.13)	6.00 %	49.10 (33.30, 63.75)	6.01 %	43.38 (27.75, 58.44)
ERBB2	12.1%	54.28 (31.94, 75.96)	1.43 %	-	1.27 %	-
FGFR1	13.4%	79.35 (67.72, 89.37)	5.43 %	67.64 (54.51, 79.59)	6.01 %	41.73 (27.57, 55.90)
HEY1	8.3%	71.47 (51.04, 89.44)	5.43 %	44.11 (30.21, 58.29)	8.23 %	45.82 (33.40, 57.90)
IRF2BP2	10.6%	44.79 (25.30, 64.61)	4.00 %	-	8.23 %	62.24 (46.76, 77.22)
KAT6A	10.8%	73.15 (52.33, 94.10)	6.00 %	57.95 (44.05, 70.81)	6.65 %	50.31 (37.01, 64.10)
MAP3K3	7.7%	51.21 (33.38, 70.97)	2.00 %	-	2.85 %	-
MCL1	10.9%	58.14 (42.83, 71.74)	10.86 %	39.15 (28.02, 50.29)	12.03 %	48.65 (37.86, 59.59)
MDM4	8.5%	62.41 (41.59, 82.65)	3.71 %	-	6.65 %	51.07 (34.28, 66.89)
MMP16	9.1%	51.77 (30.78, 72.99)	3.71 %	-	8.23 %	49.66 (36.58, 61.95)
MYC	17.1%	63.84 (48.76, 78.60)	7.14 %	49.59 (35.32, 63.64)	11.39 %	51.56 (39.22, 64.27)
NCSTN	9.3%	58.92 (42.22, 75.51)	6.57 %	46.00 (32.06, 61.83)	11.08 %	47.16 (33.34, 60.84)
NOTCH2	10.9%	47.47 (22.79, 72.55)	10.00 %	58.36 (47.17, 69.13)	10.13 %	52.33 (38.74, 66.45)

Supplementary Table 2b. AUC (with 95% CI) of the models trained on the copy number alteration data of breast cancer, and finetuned on the lung and liver cancers (Part b). Genes with empty results in LUAD and LIHC contain too few mutated samples, thus we didn't predict their copy number alteration.

Gene	BRCA		LUAD		LIHC	
	Mutation percentage	Slide AUC (95% CIs)	Mutation percentage	Slide AUC (95% CIs)	Mutation percentage	Slide AUC (95% CIs)
PAK1	7.9%	34.45 (10.00, 63.40)	3.43 %	-	0.63 %	-
PARP1	8.8%	39.11 (22.53, 57.77)	2.29 %	-	6.33 %	61.25 (46.38, 75.22)
PSEN2	9.0%	46.01 (25.05, 66.63)	2.29 %	-	6.65 %	61.39 (43.83, 77.68)
PTEN	6.1%	29.07 (4.08, 54.18)	1.14 %	-	3.80 %	-
PTK2	12.7%	51.02 (32.34, 68.18)	5.43 %	42.49 (29.94, 54.49)	10.13 %	39.31 (28.72, 51.69)
RAB25	8.6%	68.57 (52.79, 82.63)	7.71 %	47.34 (35.99, 58.84)	12.03 %	55.61 (43.56, 67.51)
RIT1	8.8%	49.76 (29.12, 69.10)	7.71 %	47.27 (34.80, 59.77)	11.71 %	54.11 (43.84, 65.84)
RNF43	6.5%	53.33 (33.42, 73.84)	0.86 %	-	2.85 %	-
RPS6KB1	9.0%	60.75 (45.16, 75.96)	2.57 %	-	4.43 %	-
RPS6KB2	6.8%	51.69 (33.16, 73.27)	1.43 %	-	2.53 %	-
RYR2	9.9%	64.50 (49.42, 78.62)	3.14 %	-	7.91 %	64.03 (50.49, 76.34)
SPOP	6.5%	50.28 (25.35, 75.07)	0.29 %	-	0.95 %	-
TGFB2	6.8%	61.40 (42.44, 80.99)	2.57 %	-	6.33 %	71.80 (59.80, 82.83)
USH2A	7.4%	55.32 (37.64, 73.98)	2.57 %	-	7.28 %	62.32 (46.53, 77.36)
ZNF217	8.2%	69.29 (49.83, 86.97)	4.86 %	-	1.90 %	-

Supplementary Table 3. AUC (with 95% CI) of the models trained on the pathway activity from mRNA expression data of breast cancer, and finetuned on the lung and liver cancers.

Pathway ID	BRCA		LUAD		LIHC	
	Positive percentage	Slide AUC (95% CIs)	Positive percentage	Slide AUC (95% CIs)	Positive percentage	Slide AUC (95% CIs)
PI3K	56.45 %	66.62 (54.41, 77.68)	43.43 %	53.13 (46.29, 59.68)	49.05 %	54.25 (46.53, 61.34)
p53	68.59 %	79.80 (69.59, 88.99)	54.57 %	60.22 (53.59, 66.53)	54.43 %	54.19 (46.79, 61.75)
Notch	51.29 %	55.28 (43.33, 66.94)	47.43 %	50.62 (43.68, 57.19)	51.90 %	52.89 (45.54, 59.66)
RTK	67.37 %	44.16 (32.50, 55.82)	46.29 %	57.04 (50.24, 63.57)	42.72 %	48.54 (41.08, 55.96)
Nrf2	53.11 %	60.58 (49.58, 71.57)	57.14 %	42.72 (36.34, 49.50)	52.53 %	52.37 (45.23, 59.80)
Wnt	51.75 %	61.41 (49.53, 71.49)	49.14 %	52.85 (46.17, 59.66)	56.65 %	38.74 (31.96, 45.87)
TGFb	61.00 %	55.12 (43.17, 66.82)	54.57 %	50.89 (44.01, 57.83)	53.48 %	51.09 (44.01, 58.70)
MYC	67.68 %	55.63 (42.66, 67.41)	54.86 %	56.47 (49.37, 63.50)	48.42 %	50.00 (43.01, 56.97)
Cell cycle	73.75 %	65.40 (54.29, 75.99)	40.86 %	51.78 (44.84, 58.47)	46.20 %	61.38 (54.30, 67.93)
Hippo	46.74 %	53.07 (41.96, 64.47)	48.57 %	53.30 (46.50, 59.83)	53.48 %	44.93 (38.02, 51.89)

Supplementary Table 4. AUC (with 95% CI) of the models trained on the pathway activity from copy number alteration data of breast cancer, and finetuned on the lung and liver cancers.

Pathway ID	BRCA		LUAD		LIHC	
	Positive percentage	Slide AUC (95% CIs)	Positive percentage	Slide AUC (95% CIs)	Positive percentage	Slide AUC (95% CIs)
PI3K	80.42 %	51.10 (37.30, 64.49)	90.29 %	60.10 (48.83, 71.02)	89.56 %	47.71 (33.98, 61.88)
p53	93.47 %	64.00 (34.36, 93.88)	90.00 %	43.14 (32.87, 54.19)	96.84 %	36.85 (16.37, 59.23)
Notch	29.74 %	66.78 (53.59, 79.51)	34.57 %	48.95 (42.01, 55.95)	30.70 %	57.22 (49.38, 64.79)
RTK	84.37 %	52.15 (34.95, 70.61)	69.43 %	52.32 (44.86, 59.34)	71.84 %	48.80 (40.10, 56.79)
Nrf2	57.06 %	39.12 (27.05, 52.07)	80.29 %	44.63 (35.83, 53.39)	54.75 %	58.74 (51.31, 66.01)
Wnt	37.18 %	42.28 (30.39, 54.01)	50.57 %	51.51 (44.82, 58.26)	61.08 %	50.54 (43.22, 57.93)
TGFb	65.40 %	58.46 (45.57, 70.63)	81.71 %	39.39 (29.61, 48.27)	61.71 %	50.50 (42.96, 57.65)
MYC	87.71 %	79.46 (67.05, 89.33)	86.29 %	65.75 (55.52, 74.96)	90.19 %	60.23 (47.69, 72.92)
Cell cycle	85.28 %	51.69 (38.29, 64.75)	88.57 %	50.63 (38.88, 62.22)	92.72 %	52.11 (38.73, 65.47)
Hippo	25.95 %	53.53 (39.45, 68.23)	32.86 %	47.04 (40.09, 53.86)	30.70 %	54.22 (45.86, 62.42)

Supplementary Note 1: The slide lists of breast, lung and liver cancers we used in our experiments.

The 659 slides in TCGA-BRCA:

TCGA-3C-AALI-01Z-00-DX2
TCGA-3C-AALJ-01Z-00-DX1
TCGA-3C-AALK-01Z-00-DX1
TCGA-5T-A9QA-01Z-00-DX1
TCGA-A1-A0SB-01Z-00-DX1
TCGA-A1-A0SE-01Z-00-DX1
TCGA-A1-A0SH-01Z-00-DX1
TCGA-A1-A0SI-01Z-00-DX1
TCGA-A1-A0SK-01Z-00-DX1
TCGA-A1-A0SM-01Z-00-DX1
TCGA-A1-A0SN-01Z-00-DX1
TCGA-A1-A0SP-01Z-00-DX1
TCGA-A1-A0SQ-01Z-00-DX1
TCGA-A2-A04P-01Z-00-DX1
TCGA-A2-A04Q-01Z-00-DX1
TCGA-A2-A04R-01Z-00-DX1
TCGA-A2-A04T-01Z-00-DX1
TCGA-A2-A04U-01Z-00-DX1
TCGA-A2-A04V-01Z-00-DX1
TCGA-A2-A04X-01Z-00-DX1
TCGA-A2-A04Y-01Z-00-DX1
TCGA-A2-A0CL-01Z-00-DX1
TCGA-A2-A0CM-01Z-00-DX1
TCGA-A2-A0CP-01Z-00-DX1
TCGA-A2-A0CQ-01Z-00-DX1
TCGA-A2-A0CS-01Z-00-DX1
TCGA-A2-A0CT-01Z-00-DX1
TCGA-A2-A0CU-01Z-00-DX1
TCGA-A2-A0CW-01Z-00-DX1
TCGA-A2-A0CX-01Z-00-DX1
TCGA-A2-A0D0-01Z-00-DX1
TCGA-A2-A0EO-01Z-00-DX1
TCGA-A2-A0EQ-01Z-00-DX1
TCGA-A2-A0ES-01Z-00-DX1
TCGA-A2-A0ET-01Z-00-DX1
TCGA-A2-A0EU-01Z-00-DX1
TCGA-A2-A0EV-01Z-00-DX1
TCGA-A2-A0EW-01Z-00-DX1
TCGA-A2-A0EX-01Z-00-DX1
TCGA-A2-A0EY-01Z-00-DX1
TCGA-A2-A0SU-01Z-00-DX1
TCGA-A2-A0SV-01Z-00-DX1
TCGA-A2-A0SY-01Z-00-DX1
TCGA-A2-A0T2-01Z-00-DX1
TCGA-A2-A0T4-01Z-00-DX1
TCGA-A2-A0T5-01Z-00-DX1
TCGA-A2-A0T6-01Z-00-DX1
TCGA-A2-A0T7-01Z-00-DX1
TCGA-A2-A0YC-01Z-00-DX1
TCGA-A2-A0YE-01Z-00-DX1
TCGA-A2-A0YF-01Z-00-DX1

TCGA-A2-A0YH-01Z-00-DX1
TCGA-A2-A0YI-01Z-00-DX1
TCGA-A2-A0YJ-01Z-00-DX1
TCGA-A2-A0YL-01Z-00-DX1
TCGA-A2-A0YM-01Z-00-DX1
TCGA-A2-A0YT-01Z-00-DX1
TCGA-A2-A1FV-01Z-00-DX1
TCGA-A2-A1FX-01Z-00-DX1
TCGA-A2-A1FZ-01Z-00-DX1
TCGA-A2-A1G0-01Z-00-DX1
TCGA-A2-A1G4-01Z-00-DX1
TCGA-A2-A1G6-01Z-00-DX1
TCGA-A2-A259-01Z-00-DX1
TCGA-A2-A25A-01Z-00-DX1
TCGA-A2-A25B-01Z-00-DX1
TCGA-A2-A25C-01Z-00-DX1
TCGA-A2-A25D-01Z-00-DX1
TCGA-A2-A25E-01Z-00-DX1
TCGA-A2-A3KC-01Z-00-DX1
TCGA-A2-A3XS-01Z-00-DX1
TCGA-A2-A3XT-01Z-00-DX1
TCGA-A2-A3XU-01Z-00-DX1
TCGA-A2-A3XV-01Z-00-DX1
TCGA-A2-A3XW-01Z-00-DX1
TCGA-A2-A3XX-01Z-00-DX1
TCGA-A2-A3XY-01Z-00-DX1
TCGA-A2-A3XZ-01Z-00-DX1
TCGA-A2-A3Y0-01Z-00-DX1
TCGA-A2-A4RW-01Z-00-DX1
TCGA-A2-A4RX-01Z-00-DX1
TCGA-A2-A4RY-01Z-00-DX1
TCGA-A2-A4S0-01Z-00-DX1
TCGA-A2-A4S1-01Z-00-DX1
TCGA-A2-A4S2-01Z-00-DX1
TCGA-A2-A4S3-01Z-00-DX1
TCGA-A7-A0CE-01Z-00-DX1
TCGA-A7-A0CH-01Z-00-DX1
TCGA-A7-A0DA-01Z-00-DX1
TCGA-A7-A0DB-01Z-00-DX1
TCGA-A7-A13D-01Z-00-DX1
TCGA-A7-A13E-01Z-00-DX1
TCGA-A7-A13F-01Z-00-DX1
TCGA-A7-A13G-01Z-00-DX2
TCGA-A7-A26I-01Z-00-DX1
TCGA-A7-A26J-01Z-00-DX1
TCGA-A7-A2KD-01Z-00-DX1
TCGA-A7-A4SE-01Z-00-DX1
TCGA-A7-A4SF-01Z-00-DX1
TCGA-A7-A6VV-01Z-00-DX1
TCGA-A7-A6VW-01Z-00-DX1
TCGA-A7-A6VX-01Z-00-DX1
TCGA-A7-A6VY-01Z-00-DX1
TCGA-AC-A2B8-01Z-00-DX1
TCGA-AC-A2BK-01Z-00-DX1
TCGA-AC-A2BM-01Z-00-DX1
TCGA-AC-A2FB-01Z-00-DX1

TCGA-AC-A2FF-01Z-00-DX1
TCGA-AC-A2FG-01Z-00-DX1
TCGA-AC-A2FK-01Z-00-DX1
TCGA-AC-A2FM-01Z-00-DX1
TCGA-AC-A2FO-01Z-00-DX1
TCGA-AC-A2QH-01Z-00-DX1
TCGA-AC-A2QI-01Z-00-DX1
TCGA-AC-A2QJ-01Z-00-DX1
TCGA-AC-A3OD-01Z-00-DX1
TCGA-AC-A3QP-01Z-00-DX1
TCGA-AC-A3QQ-01Z-00-DX1
TCGA-AC-A3TM-01Z-00-DX1
TCGA-AC-A3YJ-01Z-00-DX1
TCGA-AC-A5EH-01Z-00-DX1
TCGA-AC-A5XS-01Z-00-DX1
TCGA-AC-A62V-01Z-00-DX1
TCGA-AC-A62X-01Z-00-DX1
TCGA-AC-A6IV-01Z-00-DX1
TCGA-AC-A6IW-01Z-00-DX1
TCGA-AC-A6NO-01Z-00-DX1
TCGA-AC-A7VB-01Z-00-DX1
TCGA-AC-A7VC-01Z-00-DX1
TCGA-AN-A0AJ-01Z-00-DX1
TCGA-AN-A0AK-01Z-00-DX1
TCGA-AN-A0AL-01Z-00-DX1
TCGA-AN-A0AM-01Z-00-DX1
TCGA-AN-A0AR-01Z-00-DX1
TCGA-AN-A0AS-01Z-00-DX1
TCGA-AN-A0AT-01Z-00-DX1
TCGA-AN-A0FF-01Z-00-DX1
TCGA-AN-A0FJ-01Z-00-DX1
TCGA-AN-A0FK-01Z-00-DX1
TCGA-AN-A0FL-01Z-00-DX1
TCGA-AN-A0FN-01Z-00-DX1
TCGA-AN-A0FT-01Z-00-DX1
TCGA-AN-A0FV-01Z-00-DX1
TCGA-AN-A0FX-01Z-00-DX1
TCGA-AN-A0FY-01Z-00-DX1
TCGA-AN-A0FZ-01Z-00-DX1
TCGA-AN-A0XL-01Z-00-DX1
TCGA-AN-A0XN-01Z-00-DX1
TCGA-AN-A0XP-01Z-00-DX1
TCGA-AN-A0XT-01Z-00-DX1
TCGA-AN-A0XU-01Z-00-DX1
TCGA-AO-A03L-01Z-00-DX1
TCGA-AO-A03M-01Z-00-DX1
TCGA-AO-A03N-01Z-00-DX1
TCGA-AO-A03O-01Z-00-DX1
TCGA-AO-A03T-01Z-00-DX1
TCGA-AO-A03U-01Z-00-DX1
TCGA-AO-A0J3-01Z-00-DX1
TCGA-AO-A0J5-01Z-00-DX1
TCGA-AO-A0J6-01Z-00-DX1
TCGA-AO-A0J7-01Z-00-DX1
TCGA-AO-A0J8-01Z-00-DX1
TCGA-AO-A0J9-01Z-00-DX1

TCGA-AO-A0JB-01Z-00-DX1
TCGA-AO-A0JC-01Z-00-DX1
TCGA-AO-A0JD-01Z-00-DX1
TCGA-AO-A0JE-01Z-00-DX1
TCGA-AO-A0JF-01Z-00-DX1
TCGA-AO-A0JI-01Z-00-DX1
TCGA-AO-A0JJ-01Z-00-DX1
TCGA-AO-A0JL-01Z-00-DX1
TCGA-AO-A0JM-01Z-00-DX1
TCGA-AO-A126-01Z-00-DX1
TCGA-AO-A12A-01Z-00-DX1
TCGA-AO-A12B-01Z-00-DX1
TCGA-AO-A12D-01Z-00-DX1
TCGA-AO-A12H-01Z-00-DX1
TCGA-AO-A1KO-01Z-00-DX1
TCGA-AO-A1KP-01Z-00-DX1
TCGA-AO-A1KQ-01Z-00-DX1
TCGA-AO-A1KR-01Z-00-DX1
TCGA-AO-A1KS-01Z-00-DX1
TCGA-AO-A1KT-01Z-00-DX1
TCGA-AQ-A04H-01Z-00-DX1
TCGA-AQ-A04J-01Z-00-DX1
TCGA-AQ-A04L-01Z-00-DX1
TCGA-AQ-A0Y5-01Z-00-DX1
TCGA-AQ-A54N-01Z-00-DX1
TCGA-AQ-A54O-01Z-00-DX1
TCGA-AQ-A7U7-01Z-00-DX1
TCGA-AR-A0TP-01Z-00-DX1
TCGA-AR-A0TQ-01Z-00-DX1
TCGA-AR-A0TR-01Z-00-DX1
TCGA-AR-A0TT-01Z-00-DX1
TCGA-AR-A0TV-01Z-00-DX1
TCGA-AR-A0TW-01Z-00-DX1
TCGA-AR-A0TY-01Z-00-DX1
TCGA-AR-A0U1-01Z-00-DX1
TCGA-AR-A0U2-01Z-00-DX1
TCGA-AR-A0U3-01Z-00-DX1
TCGA-AR-A0U4-01Z-00-DX1
TCGA-AR-A1AH-01Z-00-DX1
TCGA-AR-A1AI-01Z-00-DX1
TCGA-AR-A1AJ-01Z-00-DX1
TCGA-AR-A1AK-01Z-00-DX1
TCGA-AR-A1AQ-01Z-00-DX1
TCGA-AR-A1AR-01Z-00-DX1
TCGA-AR-A1AU-01Z-00-DX1
TCGA-AR-A1AV-01Z-00-DX1
TCGA-AR-A1AW-01Z-00-DX1
TCGA-AR-A1AX-01Z-00-DX1
TCGA-AR-A1AY-01Z-00-DX1
TCGA-AR-A24H-01Z-00-DX1
TCGA-AR-A24K-01Z-00-DX1
TCGA-AR-A24L-01Z-00-DX1
TCGA-AR-A24M-01Z-00-DX1
TCGA-AR-A24N-01Z-00-DX1
TCGA-AR-A24Q-01Z-00-DX1
TCGA-AR-A24R-01Z-00-DX1

TCGA-AR-A24S-01Z-00-DX1
TCGA-AR-A24T-01Z-00-DX1
TCGA-AR-A24U-01Z-00-DX1
TCGA-AR-A24V-01Z-00-DX1
TCGA-AR-A24X-01Z-00-DX1
TCGA-AR-A24Z-01Z-00-DX1
TCGA-AR-A250-01Z-00-DX1
TCGA-AR-A251-01Z-00-DX1
TCGA-AR-A252-01Z-00-DX1
TCGA-AR-A254-01Z-00-DX1
TCGA-AR-A256-01Z-00-DX1
TCGA-AR-A2LE-01Z-00-DX1
TCGA-AR-A2LK-01Z-00-DX1
TCGA-AR-A2LM-01Z-00-DX1
TCGA-AR-A2LO-01Z-00-DX1
TCGA-AR-A5QN-01Z-00-DX1
TCGA-AR-A5QP-01Z-00-DX1
TCGA-B6-A0I1-01Z-00-DX1
TCGA-B6-A0I2-01Z-00-DX1
TCGA-B6-A0I5-01Z-00-DX1
TCGA-B6-A0IA-01Z-00-DX1
TCGA-B6-A0IB-01Z-00-DX1
TCGA-B6-A0IC-01Z-00-DX1
TCGA-B6-A0IE-01Z-00-DX1
TCGA-B6-A0IG-01Z-00-DX1
TCGA-B6-A0IK-01Z-00-DX1
TCGA-B6-A0IM-01Z-00-DX1
TCGA-B6-A0IN-01Z-00-DX1
TCGA-B6-A0IO-01Z-00-DX1
TCGA-B6-A0IP-01Z-00-DX1
TCGA-B6-A0RE-01Z-00-DX1
TCGA-B6-A0RG-01Z-00-DX1
TCGA-B6-A0RH-01Z-00-DX1
TCGA-B6-A0RI-01Z-00-DX1
TCGA-B6-A0RL-01Z-00-DX1
TCGA-B6-A0RM-01Z-00-DX1
TCGA-B6-A0RN-01Z-00-DX1
TCGA-B6-A0RO-01Z-00-DX1
TCGA-B6-A0RS-01Z-00-DX1
TCGA-B6-A0RT-01Z-00-DX1
TCGA-B6-A0RU-01Z-00-DX1
TCGA-B6-A0RV-01Z-00-DX1
TCGA-B6-A0WS-01Z-00-DX1
TCGA-B6-A0WT-01Z-00-DX1
TCGA-B6-A0WV-01Z-00-DX1
TCGA-B6-A0WW-01Z-00-DX1
TCGA-B6-A0WY-01Z-00-DX1
TCGA-B6-A0WZ-01Z-00-DX1
TCGA-B6-A0X0-01Z-00-DX1
TCGA-B6-A0X1-01Z-00-DX1
TCGA-B6-A0X4-01Z-00-DX1
TCGA-B6-A0X5-01Z-00-DX1
TCGA-B6-A1KC-01Z-00-DX1
TCGA-B6-A1KF-01Z-00-DX1
TCGA-B6-A1KN-01Z-00-DX1
TCGA-BH-A0AU-01Z-00-DX1

TCGA-BH-A0AV-01Z-00-DX1
TCGA-BH-A0AW-01Z-00-DX1
TCGA-BH-A0AY-01Z-00-DX1
TCGA-BH-A0AZ-01Z-00-DX1
TCGA-BH-A0B0-01Z-00-DX1
TCGA-BH-A0B3-01Z-00-DX1
TCGA-BH-A0B4-01Z-00-DX1
TCGA-BH-A0B5-01Z-00-DX1
TCGA-BH-A0B7-01Z-00-DX1
TCGA-BH-A0B9-01Z-00-DX1
TCGA-BH-A0BA-01Z-00-DX1
TCGA-BH-A0BC-01Z-00-DX1
TCGA-BH-A0BD-01Z-00-DX1
TCGA-BH-A0BF-01Z-00-DX1
TCGA-BH-A0BG-01Z-00-DX1
TCGA-BH-A0BJ-01Z-00-DX1
TCGA-BH-A0BQ-01Z-00-DX1
TCGA-BH-A0BR-01Z-00-DX1
TCGA-BH-A0BS-01Z-00-DX1
TCGA-BH-A0BT-01Z-00-DX1
TCGA-BH-A0BV-01Z-00-DX1
TCGA-BH-A0BW-01Z-00-DX1
TCGA-BH-A0BZ-01Z-00-DX1
TCGA-BH-A0C0-01Z-00-DX1
TCGA-BH-A0C1-01Z-00-DX1
TCGA-BH-A0C3-01Z-00-DX1
TCGA-BH-A0C7-01Z-00-DX1
TCGA-BH-A0DD-01Z-00-DX1
TCGA-BH-A0DE-01Z-00-DX1
TCGA-BH-A0DG-01Z-00-DX1
TCGA-BH-A0DH-01Z-00-DX1
TCGA-BH-A0DI-01Z-00-DX1
TCGA-BH-A0DK-01Z-00-DX1
TCGA-BH-A0DL-01Z-00-DX1
TCGA-BH-A0DO-01Z-00-DX1
TCGA-BH-A0DP-01Z-00-DX1
TCGA-BH-A0DQ-01Z-00-DX1
TCGA-BH-A0DT-01Z-00-DX1
TCGA-BH-A0DV-01Z-00-DX1
TCGA-BH-A0DX-01Z-00-DX1
TCGA-BH-A0DZ-01Z-00-DX1
TCGA-BH-A0E0-01Z-00-DX1
TCGA-BH-A0E1-01Z-00-DX1
TCGA-BH-A0E2-01Z-00-DX1
TCGA-BH-A0E6-01Z-00-DX1
TCGA-BH-A0E7-01Z-00-DX1
TCGA-BH-A0EB-01Z-00-DX1
TCGA-BH-A0EE-01Z-00-DX1
TCGA-BH-A0EI-01Z-00-DX1
TCGA-BH-A0GY-01Z-00-DX1
TCGA-BH-A0GZ-01Z-00-DX1
TCGA-BH-A0H0-01Z-00-DX1
TCGA-BH-A0H3-01Z-00-DX1
TCGA-BH-A0H5-01Z-00-DX1
TCGA-BH-A0H7-01Z-00-DX1
TCGA-BH-A0H9-01Z-00-DX1

TCGA-BH-A0HA-01Z-00-DX1
TCGA-BH-A0HB-01Z-00-DX1
TCGA-BH-A0HI-01Z-00-DX1
TCGA-BH-A0HQ-01Z-00-DX1
TCGA-BH-A0HU-01Z-00-DX1
TCGA-BH-A0HW-01Z-00-DX1
TCGA-BH-A0HX-01Z-00-DX1
TCGA-BH-A0HY-01Z-00-DX1
TCGA-BH-A0RX-01Z-00-DX1
TCGA-BH-A0W3-01Z-00-DX1
TCGA-BH-A0W4-01Z-00-DX1
TCGA-BH-A0W5-01Z-00-DX1
TCGA-BH-A0W7-01Z-00-DX1
TCGA-BH-A0WA-01Z-00-DX1
TCGA-BH-A18F-01Z-00-DX1
TCGA-BH-A18H-01Z-00-DX1
TCGA-BH-A18I-01Z-00-DX1
TCGA-BH-A18J-01Z-00-DX1
TCGA-BH-A18L-01Z-00-DX1
TCGA-BH-A18N-01Z-00-DX1
TCGA-BH-A18P-01Z-00-DX1
TCGA-BH-A18Q-01Z-00-DX1
TCGA-BH-A18S-01Z-00-DX1
TCGA-BH-A18T-01Z-00-DX1
TCGA-BH-A18U-01Z-00-DX1
TCGA-BH-A18V-01Z-00-DX1
TCGA-BH-A1ES-01Z-00-DX1
TCGA-BH-A1F2-01Z-00-DX1
TCGA-BH-A1F5-01Z-00-DX1
TCGA-BH-A1F6-01Z-00-DX1
TCGA-BH-A1F8-01Z-00-DX1
TCGA-BH-A1FB-01Z-00-DX1
TCGA-BH-A1FC-01Z-00-DX1
TCGA-BH-A1FE-01Z-00-DX1
TCGA-BH-A1FG-01Z-00-DX1
TCGA-BH-A1FJ-01Z-00-DX1
TCGA-BH-A1FL-01Z-00-DX1
TCGA-BH-A1FM-01Z-00-DX1
TCGA-BH-A1FN-01Z-00-DX1
TCGA-BH-A1FR-01Z-00-DX1
TCGA-BH-A1FU-01Z-00-DX1
TCGA-BH-A201-01Z-00-DX1
TCGA-BH-A202-01Z-00-DX1
TCGA-BH-A204-01Z-00-DX1
TCGA-BH-A28Q-01Z-00-DX1
TCGA-BH-A2L8-01Z-00-DX1
TCGA-BH-A5IZ-01Z-00-DX1
TCGA-C8-A12L-01Z-00-DX1
TCGA-C8-A12M-01Z-00-DX1
TCGA-C8-A12O-01Z-00-DX1
TCGA-C8-A12Q-01Z-00-DX1
TCGA-C8-A12T-01Z-00-DX1
TCGA-C8-A12U-01Z-00-DX1
TCGA-C8-A12V-01Z-00-DX1
TCGA-C8-A12W-01Z-00-DX1
TCGA-C8-A12Y-01Z-00-DX1

TCGA-C8-A12Z-01Z-00-DX1
TCGA-C8-A130-01Z-00-DX1
TCGA-C8-A131-01Z-00-DX1
TCGA-C8-A132-01Z-00-DX1
TCGA-C8-A134-01Z-00-DX1
TCGA-C8-A135-01Z-00-DX1
TCGA-C8-A1HE-01Z-00-DX1
TCGA-C8-A1HF-01Z-00-DX1
TCGA-C8-A1HG-01Z-00-DX1
TCGA-C8-A1HI-01Z-00-DX1
TCGA-C8-A1HJ-01Z-00-DX1
TCGA-C8-A1HK-01Z-00-DX1
TCGA-C8-A1HL-01Z-00-DX1
TCGA-C8-A1HM-01Z-00-DX1
TCGA-C8-A1HN-01Z-00-DX1
TCGA-C8-A1HO-01Z-00-DX1
TCGA-C8-A26V-01Z-00-DX1
TCGA-C8-A26W-01Z-00-DX1
TCGA-C8-A26X-01Z-00-DX1
TCGA-C8-A26Y-01Z-00-DX1
TCGA-C8-A26Z-01Z-00-DX1
TCGA-C8-A273-01Z-00-DX1
TCGA-C8-A274-01Z-00-DX1
TCGA-C8-A275-01Z-00-DX1
TCGA-C8-A27A-01Z-00-DX1
TCGA-C8-A27B-01Z-00-DX1
TCGA-C8-A3M8-01Z-00-DX1
TCGA-C8-A8HP-01Z-00-DX1
TCGA-C8-A8HQ-01Z-00-DX1
TCGA-C8-A8HR-01Z-00-DX1
TCGA-D8-A13Y-01Z-00-DX2
TCGA-D8-A13Z-01Z-00-DX1
TCGA-D8-A140-01Z-00-DX1
TCGA-D8-A141-01Z-00-DX1
TCGA-D8-A142-01Z-00-DX1
TCGA-D8-A143-01Z-00-DX1
TCGA-D8-A147-01Z-00-DX1
TCGA-D8-A1J8-01Z-00-DX1
TCGA-D8-A1J9-01Z-00-DX1
TCGA-D8-A1JC-01Z-00-DX2
TCGA-D8-A1JD-01Z-00-DX2
TCGA-D8-A1X6-01Z-00-DX2
TCGA-D8-A1X9-01Z-00-DX1
TCGA-D8-A1XB-01Z-00-DX2
TCGA-D8-A1XD-01Z-00-DX1
TCGA-D8-A1XF-01Z-00-DX1
TCGA-D8-A1XG-01Z-00-DX1
TCGA-D8-A1XL-01Z-00-DX1
TCGA-D8-A1XR-01Z-00-DX1
TCGA-D8-A1XS-01Z-00-DX1
TCGA-D8-A1XW-01Z-00-DX1
TCGA-D8-A1XZ-01Z-00-DX2
TCGA-D8-A1Y1-01Z-00-DX2
TCGA-D8-A1Y2-01Z-00-DX2
TCGA-D8-A1Y3-01Z-00-DX1
TCGA-D8-A27F-01Z-00-DX1

TCGA-D8-A27K-01Z-00-DX1
TCGA-D8-A27M-01Z-00-DX1
TCGA-D8-A27R-01Z-00-DX1
TCGA-D8-A27W-01Z-00-DX2
TCGA-D8-A3Z6-01Z-00-DX1
TCGA-E2-A105-01Z-00-DX1
TCGA-E2-A109-01Z-00-DX1
TCGA-E2-A10A-01Z-00-DX1
TCGA-E2-A10B-01Z-00-DX1
TCGA-E2-A10C-01Z-00-DX1
TCGA-E2-A10E-01Z-00-DX1
TCGA-E2-A14N-01Z-00-DX1
TCGA-E2-A14O-01Z-00-DX1
TCGA-E2-A14P-01Z-00-DX1
TCGA-E2-A14Q-01Z-00-DX1
TCGA-E2-A14R-01Z-00-DX1
TCGA-E2-A14S-01Z-00-DX1
TCGA-E2-A14T-01Z-00-DX1
TCGA-E2-A14U-01Z-00-DX1
TCGA-E2-A14V-01Z-00-DX1
TCGA-E2-A14W-01Z-00-DX1
TCGA-E2-A14X-01Z-00-DX1
TCGA-E2-A14Y-01Z-00-DX1
TCGA-E2-A14Z-01Z-00-DX1
TCGA-E2-A150-01Z-00-DX1
TCGA-E2-A152-01Z-00-DX1
TCGA-E2-A154-01Z-00-DX1
TCGA-E2-A155-01Z-00-DX1
TCGA-E2-A156-01Z-00-DX1
TCGA-E2-A158-01Z-00-DX1
TCGA-E2-A159-01Z-00-DX1
TCGA-E2-A15A-01Z-00-DX1
TCGA-E2-A15C-01Z-00-DX1
TCGA-E2-A15D-01Z-00-DX1
TCGA-E2-A15F-01Z-00-DX1
TCGA-E2-A15G-01Z-00-DX1
TCGA-E2-A15H-01Z-00-DX1
TCGA-E2-A15J-01Z-00-DX1
TCGA-E2-A15L-01Z-00-DX1
TCGA-E2-A15M-01Z-00-DX1
TCGA-E2-A15O-01Z-00-DX1
TCGA-E2-A15P-01Z-00-DX1
TCGA-E2-A15R-01Z-00-DX1
TCGA-E2-A15S-01Z-00-DX1
TCGA-E2-A15T-01Z-00-DX1
TCGA-E2-A1AZ-01Z-00-DX1
TCGA-E2-A1B0-01Z-00-DX1
TCGA-E2-A1B6-01Z-00-DX1
TCGA-E2-A1BD-01Z-00-DX1
TCGA-E2-A1IF-01Z-00-DX1
TCGA-E2-A1IG-01Z-00-DX1
TCGA-E2-A1II-01Z-00-DX1
TCGA-E2-A1IN-01Z-00-DX1
TCGA-E2-A1IO-01Z-00-DX1
TCGA-E2-A1L6-01Z-00-DX1
TCGA-E2-A1L7-01Z-00-DX1

TCGA-E2-A1L9-01Z-00-DX1
TCGA-E2-A1LA-01Z-00-DX1
TCGA-E2-A1LB-01Z-00-DX1
TCGA-E2-A1LE-01Z-00-DX1
TCGA-E2-A1LH-01Z-00-DX1
TCGA-E2-A1LI-01Z-00-DX1
TCGA-E2-A1LK-01Z-00-DX1
TCGA-E2-A1LL-01Z-00-DX1
TCGA-E2-A2P5-01Z-00-DX1
TCGA-E2-A2P6-01Z-00-DX1
TCGA-E2-A56Z-01Z-00-DX1
TCGA-E2-A570-01Z-00-DX1
TCGA-E2-A573-01Z-00-DX1
TCGA-E2-A574-01Z-00-DX1
TCGA-E2-A9RU-01Z-00-DX1
TCGA-E9-A1R0-01Z-00-DX1
TCGA-E9-A1R2-01Z-00-DX1
TCGA-E9-A1R7-01Z-00-DX1
TCGA-E9-A1RC-01Z-00-DX1
TCGA-E9-A1RG-01Z-00-DX1
TCGA-E9-A226-01Z-00-DX1
TCGA-E9-A227-01Z-00-DX1
TCGA-E9-A228-01Z-00-DX1
TCGA-E9-A229-01Z-00-DX1
TCGA-E9-A22A-01Z-00-DX1
TCGA-E9-A22B-01Z-00-DX1
TCGA-E9-A22D-01Z-00-DX1
TCGA-E9-A22E-01Z-00-DX1
TCGA-E9-A22G-01Z-00-DX1
TCGA-E9-A22H-01Z-00-DX1
TCGA-E9-A244-01Z-00-DX1
TCGA-E9-A245-01Z-00-DX1
TCGA-E9-A247-01Z-00-DX1
TCGA-E9-A248-01Z-00-DX1
TCGA-E9-A249-01Z-00-DX1
TCGA-E9-A24A-01Z-00-DX1
TCGA-E9-A295-01Z-00-DX1
TCGA-E9-A2JS-01Z-00-DX1
TCGA-E9-A3HO-01Z-00-DX1
TCGA-E9-A3Q9-01Z-00-DX1
TCGA-E9-A3X8-01Z-00-DX1
TCGA-E9-A54X-01Z-00-DX1
TCGA-E9-A54Y-01Z-00-DX1
TCGA-E9-A5FK-01Z-00-DX1
TCGA-E9-A5FL-01Z-00-DX1
TCGA-E9-A5UO-01Z-00-DX1
TCGA-E9-A6HE-01Z-00-DX1
TCGA-EW-A1IY-01Z-00-DX1
TCGA-EW-A1IZ-01Z-00-DX1
TCGA-EW-A1J1-01Z-00-DX1
TCGA-EW-A1J2-01Z-00-DX1
TCGA-EW-A1J3-01Z-00-DX1
TCGA-EW-A1J5-01Z-00-DX1
TCGA-EW-A1J6-01Z-00-DX1
TCGA-EW-A1OV-01Z-00-DX1
TCGA-EW-A1OW-01Z-00-DX1

TCGA-EW-A1OY-01Z-00-DX1
TCGA-EW-A1OZ-01Z-00-DX1
TCGA-EW-A1P1-01Z-00-DX1
TCGA-EW-A1P3-01Z-00-DX1
TCGA-EW-A1P4-01Z-00-DX1
TCGA-EW-A1P5-01Z-00-DX1
TCGA-EW-A1P6-01Z-00-DX1
TCGA-EW-A1P7-01Z-00-DX1
TCGA-EW-A1P8-01Z-00-DX1
TCGA-EW-A1PA-01Z-00-DX1
TCGA-EW-A1PB-01Z-00-DX1
TCGA-EW-A1PC-01Z-00-DX1
TCGA-EW-A1PE-01Z-00-DX1
TCGA-EW-A1PF-01Z-00-DX1
TCGA-EW-A1PG-01Z-00-DX1
TCGA-EW-A1PH-01Z-00-DX1
TCGA-EW-A2FR-01Z-00-DX1
TCGA-EW-A2FS-01Z-00-DX1
TCGA-EW-A2FV-01Z-00-DX1
TCGA-EW-A2FW-01Z-00-DX1
TCGA-EW-A3E8-01Z-00-DX1
TCGA-EW-A3U0-01Z-00-DX1
TCGA-EW-A423-01Z-00-DX1
TCGA-EW-A424-01Z-00-DX1
TCGA-EW-A6S9-01Z-00-DX1
TCGA-EW-A6SA-01Z-00-DX1
TCGA-EW-A6SB-01Z-00-DX1
TCGA-EW-A6SC-01Z-00-DX1
TCGA-EW-A6SD-01Z-00-DX1
TCGA-GI-A2C8-01Z-00-DX1
TCGA-GM-A2D9-01Z-00-DX1
TCGA-GM-A2DB-01Z-00-DX1
TCGA-GM-A2DC-01Z-00-DX1
TCGA-GM-A2DD-01Z-00-DX1
TCGA-GM-A2DF-01Z-00-DX1
TCGA-GM-A2DH-01Z-00-DX1
TCGA-GM-A2DI-01Z-00-DX1
TCGA-GM-A2DK-01Z-00-DX1
TCGA-GM-A2DL-01Z-00-DX1
TCGA-GM-A2DM-01Z-00-DX1
TCGA-GM-A2DN-01Z-00-DX1
TCGA-GM-A2DO-01Z-00-DX1
TCGA-GM-A3NW-01Z-00-DX1
TCGA-GM-A3NY-01Z-00-DX1
TCGA-GM-A3XG-01Z-00-DX1
TCGA-GM-A3XL-01Z-00-DX1
TCGA-GM-A3XN-01Z-00-DX1
TCGA-HN-A2NL-01Z-00-DX1
TCGA-LD-A66U-01Z-00-DX1
TCGA-LD-A7W5-01Z-00-DX1
TCGA-LD-A7W6-01Z-00-DX1
TCGA-LD-A9QF-01Z-00-DX1
TCGA-LL-A440-01Z-00-DX1
TCGA-LL-A441-01Z-00-DX1
TCGA-LL-A442-01Z-00-DX1
TCGA-LL-A50Y-01Z-00-DX1

TCGA-LL-A5YM-01Z-00-DX1
TCGA-LL-A5YN-01Z-00-DX1
TCGA-LL-A5YO-01Z-00-DX1
TCGA-LL-A5YP-01Z-00-DX1
TCGA-LL-A6FQ-01Z-00-DX1
TCGA-LL-A6FR-01Z-00-DX1
TCGA-LL-A73Y-01Z-00-DX1
TCGA-LL-A73Z-01Z-00-DX1
TCGA-LL-A740-01Z-00-DX1
TCGA-LL-A7SZ-01Z-00-DX1
TCGA-LL-A7T0-01Z-00-DX1
TCGA-LL-A8F5-01Z-00-DX1
TCGA-LL-A9Q3-01Z-00-DX1
TCGA-LQ-A4E4-01Z-00-DX1
TCGA-OK-A5Q2-01Z-00-DX1
TCGA-OL-A5D6-01Z-00-DX1
TCGA-OL-A5D7-01Z-00-DX1
TCGA-OL-A5D8-01Z-00-DX1
TCGA-OL-A5DA-01Z-00-DX1
TCGA-OL-A5RU-01Z-00-DX1
TCGA-OL-A5RV-01Z-00-DX1
TCGA-OL-A5RW-01Z-00-DX1
TCGA-OL-A5RX-01Z-00-DX1
TCGA-OL-A5RY-01Z-00-DX1
TCGA-OL-A5S0-01Z-00-DX1
TCGA-OL-A66I-01Z-00-DX1
TCGA-OL-A66K-01Z-00-DX1
TCGA-OL-A66N-01Z-00-DX1
TCGA-OL-A66O-01Z-00-DX1
TCGA-OL-A66P-01Z-00-DX1
TCGA-OL-A6VO-01Z-00-DX1
TCGA-OL-A6VQ-01Z-00-DX1
TCGA-OL-A97C-01Z-00-DX1
TCGA-PE-A5DD-01Z-00-DX1
TCGA-S3-A6ZF-01Z-00-DX1
TCGA-S3-A6ZG-01Z-00-DX1
TCGA-S3-A6ZH-01Z-00-DX1
TCGA-S3-AA0Z-01Z-00-DX1
TCGA-S3-AA10-01Z-00-DX1
TCGA-S3-AA12-01Z-00-DX1
TCGA-S3-AA14-01Z-00-DX1
TCGA-S3-AA15-01Z-00-DX1
TCGA-UL-AAZ6-01Z-00-DX1
TCGA-UU-A93S-01Z-00-DX1
TCGA-WT-AB41-01Z-00-DX1
TCGA-WT-AB44-01Z-00-DX1
TCGA-XX-A899-01Z-00-DX1
TCGA-Z7-A8R6-01Z-00-DX1

The 350 slides in TCGA-LUAD:

TCGA-35-3615-01Z-00-DX1
TCGA-35-4122-01Z-00-DX1
TCGA-35-4123-01Z-00-DX1
TCGA-35-5375-01Z-00-DX1
TCGA-38-4625-01Z-00-DX1

TCGA-38-4626-01Z-00-DX1
TCGA-38-4627-01Z-00-DX1
TCGA-38-4628-01Z-00-DX1
TCGA-38-4631-01Z-00-DX1
TCGA-38-4632-01Z-00-DX1
TCGA-38-6178-01Z-00-DX1
TCGA-38-7271-01Z-00-DX1
TCGA-38-A44F-01Z-00-DX1
TCGA-44-2655-01Z-00-DX1
TCGA-44-2656-01Z-00-DX1
TCGA-44-2657-01Z-00-DX1
TCGA-44-2659-01Z-00-DX1
TCGA-44-2662-01Z-00-DX1
TCGA-44-2665-01Z-00-DX1
TCGA-44-2666-01Z-00-DX1
TCGA-44-2668-01Z-00-DX1
TCGA-44-3396-01Z-00-DX1
TCGA-44-3398-01Z-00-DX1
TCGA-44-3918-01Z-00-DX1
TCGA-44-3919-01Z-00-DX1
TCGA-44-4112-01Z-00-DX1
TCGA-44-5643-01Z-00-DX1
TCGA-44-5644-01Z-00-DX1
TCGA-44-5645-01Z-00-DX1
TCGA-44-6145-01Z-00-DX1
TCGA-44-6146-01Z-00-DX1
TCGA-44-6148-01Z-00-DX1
TCGA-44-6774-01Z-00-DX1
TCGA-44-6775-01Z-00-DX1
TCGA-44-6776-01Z-00-DX1
TCGA-44-6777-01Z-00-DX1
TCGA-44-6778-01Z-00-DX1
TCGA-44-6779-01Z-00-DX1
TCGA-44-7659-01Z-00-DX1
TCGA-44-7660-01Z-00-DX1
TCGA-44-7661-01Z-00-DX1
TCGA-44-7662-01Z-00-DX1
TCGA-44-7667-01Z-00-DX1
TCGA-44-7669-01Z-00-DX1
TCGA-44-7670-01Z-00-DX1
TCGA-44-7671-01Z-00-DX1
TCGA-44-7672-01Z-00-DX1
TCGA-44-8119-01Z-00-DX1
TCGA-44-A479-01Z-00-DX1
TCGA-44-A47A-01Z-00-DX1
TCGA-44-A47B-01Z-00-DX1
TCGA-44-A47G-01Z-00-DX1
TCGA-44-A4SS-01Z-00-DX1
TCGA-44-A4SU-01Z-00-DX1
TCGA-49-4487-01Z-00-DX1
TCGA-49-4488-01Z-00-DX1
TCGA-49-4490-01Z-00-DX2
TCGA-49-4494-01Z-00-DX3
TCGA-49-4501-01Z-00-DX1
TCGA-49-4505-01Z-00-DX2
TCGA-49-4506-01Z-00-DX1

TCGA-49-4507-01Z-00-DX2
TCGA-49-4512-01Z-00-DX1
TCGA-49-4514-01Z-00-DX1
TCGA-49-6742-01Z-00-DX2
TCGA-49-6743-01Z-00-DX2
TCGA-49-6745-01Z-00-DX3
TCGA-49-6761-01Z-00-DX1
TCGA-49-6767-01Z-00-DX1
TCGA-49-AAQV-01Z-00-DX1
TCGA-49-AAR0-01Z-00-DX1
TCGA-49-AAR2-01Z-00-DX1
TCGA-49-AAR3-01Z-00-DX1
TCGA-49-AAR9-01Z-00-DX1
TCGA-49-AARE-01Z-00-DX1
TCGA-49-AARN-01Z-00-DX1
TCGA-49-AARO-01Z-00-DX1
TCGA-49-AARQ-01Z-00-DX1
TCGA-49-AARR-01Z-00-DX1
TCGA-4B-A93V-01Z-00-DX1
TCGA-50-5044-01Z-00-DX1
TCGA-50-5045-01Z-00-DX1
TCGA-50-5055-01Z-00-DX2
TCGA-50-5066-01Z-00-DX1
TCGA-50-5068-01Z-00-DX2
TCGA-50-5931-01Z-00-DX1
TCGA-50-5939-01Z-00-DX1
TCGA-50-5942-01Z-00-DX1
TCGA-50-6591-01Z-00-DX1
TCGA-50-6593-01Z-00-DX1
TCGA-50-6594-01Z-00-DX1
TCGA-50-6597-01Z-00-DX1
TCGA-50-6673-01Z-00-DX1
TCGA-50-7109-01Z-00-DX1
TCGA-50-8457-01Z-00-DX1
TCGA-50-8459-01Z-00-DX1
TCGA-53-7624-01Z-00-DX1
TCGA-53-7626-01Z-00-DX1
TCGA-53-7813-01Z-00-DX1
TCGA-53-A4EZ-01Z-00-DX1
TCGA-55-1594-01Z-00-DX1
TCGA-55-5899-01Z-00-DX1
TCGA-55-6543-01Z-00-DX1
TCGA-55-6642-01Z-00-DX1
TCGA-55-6968-01Z-00-DX1
TCGA-55-6969-01Z-00-DX1
TCGA-55-6970-01Z-00-DX1
TCGA-55-6971-01Z-00-DX1
TCGA-55-6972-01Z-00-DX1
TCGA-55-6975-01Z-00-DX1
TCGA-55-6978-01Z-00-DX1
TCGA-55-6979-01Z-00-DX1
TCGA-55-6980-01Z-00-DX1
TCGA-55-6981-01Z-00-DX1
TCGA-55-6982-01Z-00-DX1
TCGA-55-6983-01Z-00-DX1
TCGA-55-6984-01Z-00-DX1

TCGA-55-6985-01Z-00-DX1
TCGA-55-6986-01Z-00-DX1
TCGA-55-6987-01Z-00-DX1
TCGA-55-7281-01Z-00-DX1
TCGA-55-7283-01Z-00-DX1
TCGA-55-7570-01Z-00-DX1
TCGA-55-7573-01Z-00-DX1
TCGA-55-7574-01Z-00-DX1
TCGA-55-7576-01Z-00-DX1
TCGA-55-7724-01Z-00-DX1
TCGA-55-7725-01Z-00-DX1
TCGA-55-7726-01Z-00-DX1
TCGA-55-7728-01Z-00-DX1
TCGA-55-7815-01Z-00-DX1
TCGA-55-7903-01Z-00-DX1
TCGA-55-7907-01Z-00-DX1
TCGA-55-7910-01Z-00-DX1
TCGA-55-7911-01Z-00-DX1
TCGA-55-7913-01Z-00-DX1
TCGA-55-7914-01Z-00-DX1
TCGA-55-7994-01Z-00-DX1
TCGA-55-7995-01Z-00-DX1
TCGA-55-8085-01Z-00-DX1
TCGA-55-8087-01Z-00-DX1
TCGA-55-8089-01Z-00-DX1
TCGA-55-8090-01Z-00-DX1
TCGA-55-8091-01Z-00-DX1
TCGA-55-8092-01Z-00-DX1
TCGA-55-8094-01Z-00-DX1
TCGA-55-8096-01Z-00-DX1
TCGA-55-8097-01Z-00-DX1
TCGA-55-8203-01Z-00-DX1
TCGA-55-8205-01Z-00-DX1
TCGA-55-8206-01Z-00-DX1
TCGA-55-8208-01Z-00-DX1
TCGA-55-8299-01Z-00-DX1
TCGA-55-8301-01Z-00-DX1
TCGA-55-8302-01Z-00-DX1
TCGA-55-8505-01Z-00-DX1
TCGA-55-8506-01Z-00-DX1
TCGA-55-8507-01Z-00-DX1
TCGA-55-8508-01Z-00-DX1
TCGA-55-8510-01Z-00-DX1
TCGA-55-8511-01Z-00-DX1
TCGA-55-8512-01Z-00-DX1
TCGA-55-8514-01Z-00-DX1
TCGA-55-8614-01Z-00-DX1
TCGA-55-8615-01Z-00-DX1
TCGA-55-8616-01Z-00-DX1
TCGA-55-8619-01Z-00-DX1
TCGA-55-8620-01Z-00-DX1
TCGA-55-8621-01Z-00-DX1
TCGA-55-A48X-01Z-00-DX1
TCGA-55-A48Y-01Z-00-DX1
TCGA-55-A48Z-01Z-00-DX1
TCGA-55-A490-01Z-00-DX1

TCGA-55-A491-01Z-00-DX1
TCGA-55-A493-01Z-00-DX1
TCGA-55-A494-01Z-00-DX1
TCGA-55-A4DF-01Z-00-DX1
TCGA-55-A4DG-01Z-00-DX1
TCGA-55-A57B-01Z-00-DX1
TCGA-62-A46O-01Z-00-DX1
TCGA-62-A46P-01Z-00-DX1
TCGA-62-A46R-01Z-00-DX1
TCGA-62-A470-01Z-00-DX1
TCGA-62-A471-01Z-00-DX1
TCGA-62-A472-01Z-00-DX1
TCGA-64-1681-01Z-00-DX1
TCGA-64-5775-01Z-00-DX1
TCGA-64-5779-01Z-00-DX1
TCGA-64-5781-01Z-00-DX1
TCGA-64-5815-01Z-00-DX1
TCGA-67-4679-01Z-00-DX1
TCGA-67-6215-01Z-00-DX1
TCGA-67-6216-01Z-00-DX1
TCGA-67-6217-01Z-00-DX1
TCGA-69-7760-01Z-00-DX1
TCGA-69-7761-01Z-00-DX1
TCGA-69-7763-01Z-00-DX1
TCGA-69-7764-01Z-00-DX1
TCGA-69-7765-01Z-00-DX1
TCGA-69-7973-01Z-00-DX1
TCGA-69-7974-01Z-00-DX1
TCGA-69-7978-01Z-00-DX1
TCGA-69-7979-01Z-00-DX1
TCGA-69-8253-01Z-00-DX1
TCGA-69-8254-01Z-00-DX1
TCGA-69-8255-01Z-00-DX1
TCGA-69-8453-01Z-00-DX1
TCGA-69-A59K-01Z-00-DX1
TCGA-73-4658-01Z-00-DX1
TCGA-73-4659-01Z-00-DX1
TCGA-73-4662-01Z-00-DX1
TCGA-73-4666-01Z-00-DX1
TCGA-73-4668-01Z-00-DX1
TCGA-73-4670-01Z-00-DX1
TCGA-73-4675-01Z-00-DX1
TCGA-73-4676-01Z-00-DX1
TCGA-73-4677-01Z-00-DX1
TCGA-73-7498-01Z-00-DX1
TCGA-73-7499-01Z-00-DX1
TCGA-73-A9RS-01Z-00-DX1
TCGA-75-5122-01Z-00-DX1
TCGA-75-5125-01Z-00-DX1
TCGA-75-5126-01Z-00-DX1
TCGA-75-5146-01Z-00-DX1
TCGA-75-5147-01Z-00-DX1
TCGA-75-6205-01Z-00-DX1
TCGA-75-6211-01Z-00-DX1
TCGA-75-6212-01Z-00-DX1
TCGA-75-6214-01Z-00-DX1

TCGA-75-7025-01Z-00-DX1
TCGA-75-7027-01Z-00-DX1
TCGA-75-7030-01Z-00-DX1
TCGA-75-7031-01Z-00-DX1
TCGA-78-7145-01Z-00-DX1
TCGA-78-7146-01Z-00-DX1
TCGA-78-7147-01Z-00-DX1
TCGA-78-7148-01Z-00-DX1
TCGA-78-7150-01Z-00-DX1
TCGA-78-7152-01Z-00-DX1
TCGA-78-7153-01Z-00-DX1
TCGA-78-7154-01Z-00-DX1
TCGA-78-7155-01Z-00-DX1
TCGA-78-7156-01Z-00-DX1
TCGA-78-7159-01Z-00-DX1
TCGA-78-7160-01Z-00-DX1
TCGA-78-7161-01Z-00-DX1
TCGA-78-7220-01Z-00-DX1
TCGA-78-7535-01Z-00-DX1
TCGA-78-7536-01Z-00-DX1
TCGA-78-7537-01Z-00-DX1
TCGA-78-7539-01Z-00-DX1
TCGA-78-7542-01Z-00-DX1
TCGA-78-7633-01Z-00-DX1
TCGA-78-8648-01Z-00-DX1
TCGA-78-8655-01Z-00-DX1
TCGA-78-8660-01Z-00-DX1
TCGA-78-8662-01Z-00-DX1
TCGA-80-5607-01Z-00-DX1
TCGA-80-5608-01Z-00-DX1
TCGA-80-5611-01Z-00-DX1
TCGA-83-5908-01Z-00-DX1
TCGA-86-7701-01Z-00-DX1
TCGA-86-7711-01Z-00-DX1
TCGA-86-7713-01Z-00-DX1
TCGA-86-7714-01Z-00-DX1
TCGA-86-7953-01Z-00-DX1
TCGA-86-8055-01Z-00-DX1
TCGA-86-8056-01Z-00-DX1
TCGA-86-8073-01Z-00-DX1
TCGA-86-8074-01Z-00-DX1
TCGA-86-8075-01Z-00-DX1
TCGA-86-8278-01Z-00-DX1
TCGA-86-8279-01Z-00-DX1
TCGA-86-8280-01Z-00-DX1
TCGA-86-8281-01Z-00-DX1
TCGA-86-8358-01Z-00-DX1
TCGA-86-8585-01Z-00-DX1
TCGA-86-8668-01Z-00-DX1
TCGA-86-8669-01Z-00-DX1
TCGA-86-8672-01Z-00-DX1
TCGA-86-8673-01Z-00-DX1
TCGA-86-8674-01Z-00-DX1
TCGA-86-A4D0-01Z-00-DX1
TCGA-86-A4JF-01Z-00-DX1
TCGA-86-A4P8-01Z-00-DX1

TCGA-91-A4BC-01Z-00-DX1
TCGA-91-A4BD-01Z-00-DX1
TCGA-93-7347-01Z-00-DX1
TCGA-93-8067-01Z-00-DX1
TCGA-93-A4JN-01Z-00-DX1
TCGA-93-A4JO-01Z-00-DX1
TCGA-93-A4JP-01Z-00-DX1
TCGA-93-A4JQ-01Z-00-DX1
TCGA-97-7547-01Z-00-DX1
TCGA-97-7552-01Z-00-DX1
TCGA-97-7554-01Z-00-DX1
TCGA-97-7937-01Z-00-DX1
TCGA-97-7938-01Z-00-DX1
TCGA-97-7941-01Z-00-DX1
TCGA-97-8172-01Z-00-DX1
TCGA-97-8175-01Z-00-DX1
TCGA-97-8177-01Z-00-DX1
TCGA-97-8552-01Z-00-DX1
TCGA-97-A4M3-01Z-00-DX1
TCGA-97-A4M5-01Z-00-DX1
TCGA-97-A4M7-01Z-00-DX2
TCGA-99-7458-01Z-00-DX1
TCGA-99-8025-01Z-00-DX1
TCGA-99-8028-01Z-00-DX1
TCGA-99-8033-01Z-00-DX1
TCGA-99-AA5R-01Z-00-DX1
TCGA-J2-8192-01Z-00-DX1
TCGA-J2-8194-01Z-00-DX1
TCGA-J2-A4AG-01Z-00-DX1
TCGA-L4-A4E5-01Z-00-DX1
TCGA-L4-A4E6-01Z-00-DX1
TCGA-L9-A443-01Z-00-DX1
TCGA-L9-A444-01Z-00-DX1
TCGA-L9-A50W-01Z-00-DX1
TCGA-L9-A5IP-01Z-00-DX1
TCGA-L9-A743-01Z-00-DX1
TCGA-L9-A7SV-01Z-00-DX1
TCGA-L9-A8F4-01Z-00-DX1
TCGA-MN-A4N1-01Z-00-DX1
TCGA-MN-A4N4-01Z-00-DX1
TCGA-MN-A4N5-01Z-00-DX1
TCGA-MP-A4SV-01Z-00-DX1
TCGA-MP-A4SW-01Z-00-DX1
TCGA-MP-A4SY-01Z-00-DX1
TCGA-MP-A4T4-01Z-00-DX1
TCGA-MP-A4T6-01Z-00-DX1
TCGA-MP-A4T7-01Z-00-DX1
TCGA-MP-A4T8-01Z-00-DX1
TCGA-MP-A4T9-01Z-00-DX1
TCGA-MP-A4TA-01Z-00-DX1
TCGA-MP-A4TC-01Z-00-DX1
TCGA-MP-A4TD-01Z-00-DX1
TCGA-MP-A4TE-01Z-00-DX1
TCGA-MP-A4TF-01Z-00-DX1
TCGA-MP-A4TH-01Z-00-DX1
TCGA-MP-A4TI-01Z-00-DX1

TCGA-MP-A4TJ-01Z-00-DX1
TCGA-MP-A4TK-01Z-00-DX1
TCGA-MP-A5C7-01Z-00-DX1
TCGA-NJ-A4YF-01Z-00-DX1
TCGA-NJ-A4YG-01Z-00-DX1
TCGA-NJ-A4YQ-01Z-00-DX1
TCGA-NJ-A55A-01Z-00-DX1
TCGA-NJ-A55R-01Z-00-DX1
TCGA-S2-AA1A-01Z-00-DX1

The 316 slides in TCGA-LIHC:

TCGA-2Y-A9GS-01Z-00-DX1
TCGA-2Y-A9GT-01Z-00-DX1
TCGA-2Y-A9GU-01Z-00-DX1
TCGA-2Y-A9GV-01Z-00-DX1
TCGA-2Y-A9GW-01Z-00-DX1
TCGA-2Y-A9GY-01Z-00-DX1
TCGA-2Y-A9GZ-01Z-00-DX1
TCGA-2Y-A9H0-01Z-00-DX1
TCGA-2Y-A9H1-01Z-00-DX1
TCGA-2Y-A9H2-01Z-00-DX1
TCGA-2Y-A9H3-01Z-00-DX1
TCGA-2Y-A9H4-01Z-00-DX1
TCGA-2Y-A9H5-01Z-00-DX1
TCGA-2Y-A9H6-01Z-00-DX1
TCGA-2Y-A9H7-01Z-00-DX1
TCGA-2Y-A9H8-01Z-00-DX1
TCGA-2Y-A9H9-01Z-00-DX1
TCGA-2Y-A9HA-01Z-00-DX1
TCGA-2Y-A9HB-01Z-00-DX1
TCGA-4R-AA8I-01Z-00-DX1
TCGA-5C-A9VG-01Z-00-DX1
TCGA-5C-A9VH-01Z-00-DX1
TCGA-5C-AAPD-01Z-00-DX1
TCGA-5R-AA1C-01Z-00-DX1
TCGA-5R-AA1D-01Z-00-DX1
TCGA-5R-AAAM-01Z-00-DX1
TCGA-BC-4072-01Z-00-DX1
TCGA-BC-4073-01Z-00-DX1
TCGA-BC-A10R-01Z-00-DX1
TCGA-BC-A10S-01Z-00-DX1
TCGA-BC-A10T-01Z-00-DX1
TCGA-BC-A10U-01Z-00-DX1
TCGA-BC-A10W-01Z-00-DX1
TCGA-BC-A10X-01Z-00-DX1
TCGA-BC-A10Y-01Z-00-DX1
TCGA-BC-A10Z-01Z-00-DX1
TCGA-BC-A110-01Z-00-DX1
TCGA-BC-A112-01Z-00-DX1
TCGA-BC-A216-01Z-00-DX1
TCGA-BC-A3KF-01Z-00-DX1
TCGA-BC-A3KG-01Z-00-DX1
TCGA-BC-A5W4-01Z-00-DX1
TCGA-BC-A69H-01Z-00-DX1

TCGA-BC-A69I-01Z-00-DX1
TCGA-BC-A8YO-01Z-00-DX1
TCGA-BW-A5NO-01Z-00-DX1
TCGA-BW-A5NP-01Z-00-DX1
TCGA-BW-A5NQ-01Z-00-DX1
TCGA-CC-5258-01Z-00-DX1
TCGA-CC-5260-01Z-00-DX1
TCGA-CC-5261-01Z-00-DX1
TCGA-CC-5262-01Z-00-DX1
TCGA-CC-5263-01Z-00-DX1
TCGA-CC-5264-01Z-00-DX1
TCGA-CC-A3M9-01Z-00-DX1
TCGA-CC-A3MA-01Z-00-DX1
TCGA-CC-A3MB-01Z-00-DX1
TCGA-CC-A3MC-01Z-00-DX1
TCGA-CC-A5UC-01Z-00-DX1
TCGA-CC-A5UD-01Z-00-DX1
TCGA-CC-A5UE-01Z-00-DX1
TCGA-CC-A7IE-01Z-00-DX1
TCGA-CC-A7IF-01Z-00-DX1
TCGA-CC-A7IG-01Z-00-DX1
TCGA-CC-A7IH-01Z-00-DX1
TCGA-CC-A7II-01Z-00-DX1
TCGA-CC-A7IJ-01Z-00-DX1
TCGA-CC-A7IK-01Z-00-DX1
TCGA-CC-A7IL-01Z-00-DX1
TCGA-CC-A8HT-01Z-00-DX1
TCGA-CC-A8HU-01Z-00-DX1
TCGA-CC-A8HV-01Z-00-DX1
TCGA-CC-A9FS-01Z-00-DX1
TCGA-DD-A113-01Z-00-DX1
TCGA-DD-A114-01Z-00-DX1
TCGA-DD-A116-01Z-00-DX1
TCGA-DD-A118-01Z-00-DX1
TCGA-DD-A119-01Z-00-DX1
TCGA-DD-A11A-01Z-00-DX1
TCGA-DD-A11B-01Z-00-DX1
TCGA-DD-A11C-01Z-00-DX1
TCGA-DD-A11D-01Z-00-DX1
TCGA-DD-A1EA-01Z-00-DX1
TCGA-DD-A1EB-01Z-00-DX1
TCGA-DD-A1EC-01Z-00-DX1
TCGA-DD-A1ED-01Z-00-DX1
TCGA-DD-A1EE-01Z-00-DX1
TCGA-DD-A1EF-01Z-00-DX1
TCGA-DD-A1EG-01Z-00-DX1
TCGA-DD-A1EH-01Z-00-DX1
TCGA-DD-A1EI-01Z-00-DX1
TCGA-DD-A1EJ-01Z-00-DX1
TCGA-DD-A1EK-01Z-00-DX1
TCGA-DD-A1EL-01Z-00-DX1
TCGA-DD-A39V-01Z-00-DX1
TCGA-DD-A39W-01Z-00-DX1
TCGA-DD-A39X-01Z-00-DX1
TCGA-DD-A39Y-01Z-00-DX1
TCGA-DD-A39Z-01Z-00-DX1

TCGA-DD-A3A1-01Z-00-DX1
TCGA-DD-A3A2-01Z-00-DX1
TCGA-DD-A3A3-01Z-00-DX1
TCGA-DD-A3A4-01Z-00-DX1
TCGA-DD-A3A5-01Z-00-DX1
TCGA-DD-A3A6-01Z-00-DX1
TCGA-DD-A3A7-01Z-00-DX1
TCGA-DD-A3A8-01Z-00-DX1
TCGA-DD-A3A9-01Z-00-DX1
TCGA-DD-A4NA-01Z-00-DX1
TCGA-DD-A4NB-01Z-00-DX1
TCGA-DD-A4ND-01Z-00-DX1
TCGA-DD-A4NF-01Z-00-DX1
TCGA-DD-A4NH-01Z-00-DX1
TCGA-DD-A4NI-01Z-00-DX1
TCGA-DD-A4NJ-01Z-00-DX1
TCGA-DD-A4NK-01Z-00-DX1
TCGA-DD-A4NL-01Z-00-DX1
TCGA-DD-A4NN-01Z-00-DX1
TCGA-DD-A4NO-01Z-00-DX1
TCGA-DD-A4NP-01Z-00-DX1
TCGA-DD-A4NQ-01Z-00-DX1
TCGA-DD-A4NR-01Z-00-DX1
TCGA-DD-A4NS-01Z-00-DX1
TCGA-DD-A4NV-01Z-00-DX1
TCGA-DD-A73A-01Z-00-DX2
TCGA-DD-A73B-01Z-00-DX2
TCGA-DD-A73D-01Z-00-DX2
TCGA-DD-A73E-01Z-00-DX2
TCGA-DD-A73F-01Z-00-DX1
TCGA-DD-A73G-01Z-00-DX2
TCGA-DD-AA3A-01Z-00-DX1
TCGA-DD-AAC8-01Z-00-DX1
TCGA-DD-AAC9-01Z-00-DX1
TCGA-DD-AACA-01Z-00-DX1
TCGA-DD-AACB-01Z-00-DX1
TCGA-DD-AACC-01Z-00-DX1
TCGA-DD-AACD-01Z-00-DX1
TCGA-DD-AACE-01Z-00-DX1
TCGA-DD-AACF-01Z-00-DX1
TCGA-DD-AACG-01Z-00-DX1
TCGA-DD-AACH-01Z-00-DX1
TCGA-DD-AACI-01Z-00-DX1
TCGA-DD-AACJ-01Z-00-DX1
TCGA-DD-AACK-01Z-00-DX1
TCGA-DD-AACL-01Z-00-DX1
TCGA-DD-AACN-01Z-00-DX1
TCGA-DD-AACO-01Z-00-DX1
TCGA-DD-AACP-01Z-00-DX1
TCGA-DD-AACQ-01Z-00-DX1
TCGA-DD-AACS-01Z-00-DX1
TCGA-DD-AACT-01Z-00-DX1
TCGA-DD-AACU-01Z-00-DX1
TCGA-DD-AACV-01Z-00-DX1
TCGA-DD-AACW-01Z-00-DX1
TCGA-DD-AACX-01Z-00-DX1

TCGA-DD-AACY-01Z-00-DX1
TCGA-DD-AACZ-01Z-00-DX1
TCGA-DD-AAD1-01Z-00-DX1
TCGA-DD-AAD2-01Z-00-DX1
TCGA-DD-AAD3-01Z-00-DX1
TCGA-DD-AAD6-01Z-00-DX1
TCGA-DD-AAD8-01Z-00-DX1
TCGA-DD-AADA-01Z-00-DX1
TCGA-DD-AADB-01Z-00-DX1
TCGA-DD-AADC-01Z-00-DX1
TCGA-DD-AADD-01Z-00-DX1
TCGA-DD-AADF-01Z-00-DX1
TCGA-DD-AADG-01Z-00-DX1
TCGA-DD-AADI-01Z-00-DX1
TCGA-DD-AADK-01Z-00-DX1
TCGA-DD-AADL-01Z-00-DX1
TCGA-DD-AADM-01Z-00-DX1
TCGA-DD-AADN-01Z-00-DX1
TCGA-DD-AADO-01Z-00-DX1
TCGA-DD-AADP-01Z-00-DX1
TCGA-DD-AADQ-01Z-00-DX1
TCGA-DD-AADR-01Z-00-DX1
TCGA-DD-AADS-01Z-00-DX1
TCGA-DD-AADU-01Z-00-DX1
TCGA-DD-AADV-01Z-00-DX1
TCGA-DD-AADW-01Z-00-DX1
TCGA-DD-AADY-01Z-00-DX1
TCGA-DD-AAE0-01Z-00-DX1
TCGA-DD-AAE1-01Z-00-DX1
TCGA-DD-AAE2-01Z-00-DX1
TCGA-DD-AAE4-01Z-00-DX1
TCGA-DD-AAE6-01Z-00-DX1
TCGA-DD-AAE9-01Z-00-DX1
TCGA-DD-AAEA-01Z-00-DX1
TCGA-DD-AAEB-01Z-00-DX1
TCGA-DD-AAED-01Z-00-DX1
TCGA-DD-AAEE-01Z-00-DX1
TCGA-DD-AAEG-01Z-00-DX1
TCGA-DD-AAEH-01Z-00-DX1
TCGA-DD-AAEI-01Z-00-DX1
TCGA-DD-AAVP-01Z-00-DX1
TCGA-DD-AAVQ-01Z-00-DX1
TCGA-DD-AAVR-01Z-00-DX1
TCGA-DD-AAVS-01Z-00-DX1
TCGA-DD-AAVU-01Z-00-DX1
TCGA-DD-AAVV-01Z-00-DX1
TCGA-DD-AAVW-01Z-00-DX1
TCGA-DD-AAVX-01Z-00-DX1
TCGA-DD-AAVY-01Z-00-DX1
TCGA-DD-AAVZ-01Z-00-DX1
TCGA-DD-AAW0-01Z-00-DX1
TCGA-DD-AAW1-01Z-00-DX1
TCGA-DD-AAW2-01Z-00-DX1
TCGA-DD-AAW3-01Z-00-DX1
TCGA-ED-A459-01Z-00-DX1
TCGA-ED-A4XI-01Z-00-DX1

TCGA-ED-A5KG-01Z-00-DX1
TCGA-ED-A627-01Z-00-DX1
TCGA-ED-A66X-01Z-00-DX1
TCGA-ED-A66Y-01Z-00-DX1
TCGA-ED-A7PY-01Z-00-DX1
TCGA-ED-A7XP-01Z-00-DX1
TCGA-ED-A82E-01Z-00-DX1
TCGA-ED-A8O5-01Z-00-DX1
TCGA-ED-A8O6-01Z-00-DX1
TCGA-EP-A12J-01Z-00-DX1
TCGA-EP-A26S-01Z-00-DX1
TCGA-EP-A2KB-01Z-00-DX1
TCGA-EP-A3JL-01Z-00-DX1
TCGA-EP-A3RK-01Z-00-DX1
TCGA-FV-A23B-01Z-00-DX1
TCGA-FV-A2QR-01Z-00-DX1
TCGA-FV-A3I0-01Z-00-DX1
TCGA-FV-A3I1-01Z-00-DX1
TCGA-FV-A3R2-01Z-00-DX1
TCGA-FV-A3R3-01Z-00-DX1
TCGA-FV-A495-01Z-00-DX1
TCGA-FV-A496-01Z-00-DX1
TCGA-G3-A25S-01Z-00-DX1
TCGA-G3-A25T-01Z-00-DX1
TCGA-G3-A25U-01Z-00-DX1
TCGA-G3-A25V-01Z-00-DX1
TCGA-G3-A25X-01Z-00-DX1
TCGA-G3-A25Y-01Z-00-DX1
TCGA-G3-A25Z-01Z-00-DX1
TCGA-G3-A3CH-01Z-00-DX1
TCGA-G3-A3CI-01Z-00-DX1
TCGA-G3-A3CJ-01Z-00-DX1
TCGA-G3-A3CK-01Z-00-DX1
TCGA-G3-A5SJ-01Z-00-DX1
TCGA-G3-A5SL-01Z-00-DX1
TCGA-G3-A5SM-01Z-00-DX1
TCGA-G3-A6UC-01Z-00-DX1
TCGA-G3-A7M5-01Z-00-DX1
TCGA-G3-A7M6-01Z-00-DX1
TCGA-G3-A7M7-01Z-00-DX1
TCGA-G3-A7M8-01Z-00-DX1
TCGA-G3-AAUZ-01Z-00-DX1
TCGA-G3-AAV0-01Z-00-DX1
TCGA-G3-AAV1-01Z-00-DX1
TCGA-G3-AAV2-01Z-00-DX1
TCGA-G3-AAV3-01Z-00-DX1
TCGA-G3-AAV4-01Z-00-DX1
TCGA-G3-AAV5-01Z-00-DX1
TCGA-G3-AAV6-01Z-00-DX1
TCGA-G3-AAV7-01Z-00-DX1
TCGA-GJ-A3OU-01Z-00-DX1
TCGA-GJ-A9DB-01Z-00-DX1
TCGA-HP-A5MZ-01Z-00-DX1
TCGA-HP-A5N0-01Z-00-DX1
TCGA-K7-A5RG-01Z-00-DX1
TCGA-K7-A6G5-01Z-00-DX1

TCGA-K7-AAU7-01Z-00-DX1
TCGA-LG-A6GG-01Z-00-DX1
TCGA-LG-A9QC-01Z-00-DX1
TCGA-LG-A9QD-01Z-00-DX1
TCGA-MI-A75C-01Z-00-DX1
TCGA-MI-A75E-01Z-00-DX1
TCGA-MI-A75G-01Z-00-DX1
TCGA-MI-A75H-01Z-00-DX1
TCGA-MI-A75I-01Z-00-DX1
TCGA-MR-A8JO-01Z-00-DX1
TCGA-NI-A4U2-01Z-00-DX1
TCGA-NI-A8LF-01Z-00-DX1
TCGA-O8-A75V-01Z-00-DX1
TCGA-PD-A5DF-01Z-00-DX1
TCGA-RC-A6M3-01Z-00-DX1
TCGA-RC-A6M6-01Z-00-DX1
TCGA-RC-A7S9-01Z-00-DX1
TCGA-RC-A7SH-01Z-00-DX1
TCGA-RC-A7SK-01Z-00-DX1
TCGA-RG-A7D4-01Z-00-DX1
TCGA-UB-A7MA-01Z-00-DX1
TCGA-UB-A7MB-01Z-00-DX1
TCGA-UB-A7MC-01Z-00-DX1
TCGA-UB-A7MD-01Z-00-DX1
TCGA-UB-A7ME-01Z-00-DX1
TCGA-UB-A7MF-01Z-00-DX1
TCGA-UB-AA0U-01Z-00-DX1
TCGA-UB-AA0V-01Z-00-DX1
TCGA-WJ-A86L-01Z-00-DX1
TCGA-WQ-A9G7-01Z-00-DX1
TCGA-WQ-AB4B-01Z-00-DX1
TCGA-WX-AA44-01Z-00-DX1
TCGA-WX-AA47-01Z-00-DX1
TCGA-XR-A8TD-01Z-00-DX1
TCGA-XR-A8TE-01Z-00-DX1
TCGA-XR-A8TF-01Z-00-DX1
TCGA-XR-A8TG-01Z-00-DX1
TCGA-YA-A8S7-01Z-00-DX1
TCGA-ZP-A9CV-01Z-00-DX1
TCGA-ZP-A9CY-01Z-00-DX1
TCGA-ZP-A9CZ-01Z-00-DX1
TCGA-ZP-A9D0-01Z-00-DX1
TCGA-ZP-A9D1-01Z-00-DX1
TCGA-ZP-A9D2-01Z-00-DX1
TCGA-ZP-A9D4-01Z-00-DX1
TCGA-ZS-A9CD-01Z-00-DX1
TCGA-ZS-A9CE-01Z-00-DX1
TCGA-ZS-A9CF-01Z-00-DX1
TCGA-ZS-A9CG-01Z-00-DX1

Supplementary Note 2: links to download data

Point mutation of 18 genes (download the Sample matrix in the following page):
Breast cancer:
https://www.cbioportal.org/results/download?cancer_study_list=brca_tcga_pan_can_atlas_2018&Z_SCO
RE_THRESHOLD=2.0&RPPA_SCORE_THRESHOLD=2.0&data_priority=0&profileFilter=0&case_set_id
=brca_tcga_pan_can_atlas_2018_all&gene_list=ARID1A%250ACDH1%250ACTCF%250AERBB2%250
AGATA3%250AKMT2C%250AMAP2K4%250AMAP3K1%250ANF1%250ANOTCH2%250APIK3CA%250
APTEN%250ARB1%250ARUNX1%250ARYR2%250ATBX3%250ATP53%250AUSH2A&geneset_list=%
20&tab_index=tab_visualize&Action=Submit&genetic_profile_ids_PROFILE_MUTATION_EXTENDED=br
ca_tcga_pan_can_atlas_2018_mutations

Lung cancer:
https://www.cbioportal.org/results/download?cancer_study_list=luad_tcga_pan_can_atlas_2018&Z_SCO
RE_THRESHOLD=2.0&RPPA_SCORE_THRESHOLD=2.0&data_priority=0&profileFilter=0&case_set_id
=luad_tcga_pan_can_atlas_2018_all&gene_list=ARID1A%250ACDH1%250ACTCF%250AERBB2%250A
GATA3%250AKMT2C%250AMAP2K4%250AMAP3K1%250ANF1%250ANOTCH2%250APIK3CA%250A
PTEN%250ARB1%250ARUNX1%250ARYR2%250ATBX3%250ATP53%250AUSH2A&geneset_list=%2
0&tab_index=tab_visualize&Action=Submit&genetic_profile_ids_PROFILE_MUTATION_EXTENDED=lua
d_tcga_pan_can_atlas_2018_mutations

Liver cancer:
https://www.cbioportal.org/results/download?cancer_study_list=lihc_tcga_pan_can_atlas_2018&Z_SCOR
E_THRESHOLD=2.0&RPPA_SCORE_THRESHOLD=2.0&data_priority=0&profileFilter=0&case_set_id=li
hc_tcga_pan_can_atlas_2018_all&gene_list=ARID1A%250ACDH1%250ACTCF%250AERBB2%250AGA
TA3%250AKMT2C%250AMAP2K4%250AMAP3K1%250ANF1%250ANOTCH2%250APIK3CA%250APT
EN%250ARB1%250ARUNX1%250ARYR2%250ATBX3%250ATP53%250AUSH2A&geneset_list=%20&t
ab_index=tab_visualize&Action=Submit&genetic_profile_ids_PROFILE_MUTATION_EXTENDED=lihc_tc
ga_pan_can_atlas_2018_mutations

Copy number alteration of 35 genes (download the Sample matrix in the following page):
Breast cancer:
https://www.cbioportal.org/results/download?cancer_study_list=brca_tcga_pan_can_atlas_2018&Z_SCO
RE_THRESHOLD=2.0&RPPA_SCORE_THRESHOLD=2.0&data_priority=0&profileFilter=0&case_set_id
=brca_tcga_pan_can_atlas_2018_all&gene_list=AKT3%250AAPH1A%250ABMP7%250ACCND1%250A
CDKN2A%250ACDKN2B%250AE2F5%250AEIF4EBP1%250AERBB2%250AFGFR1%250AHEY1%250
AIRF2BP2%250AKAT6A%250AMAP3K3%250AMCL1%250AMDM4%250AMMP16%250AMYC%250AN
CSTN%250ANOTCH2%250APAK1%250APARP1%250APSEN2%250APTEN%250APTK2%250ARAB2
5%250ARIT1%250ARNF43%250ARPS6KB1%250ARPS6KB2%250ARYR2%250ASPOP%250ATGFB2
%250AUSH2A%250AZNF217&geneset_list=%20&tab_index=tab_visualize&Action=Submit&genetic_prof
ile_ids_PROFILE_COPY_NUMBER_ALTERATION=brca_tcga_pan_can_atlas_2018_gistic

Lung cancer:
https://www.cbioportal.org/results/download?cancer_study_list=luad_tcga_pan_can_atlas_2018&Z_SCO
RE_THRESHOLD=2.0&RPPA_SCORE_THRESHOLD=2.0&data_priority=0&profileFilter=0&case_set_id
=luad_tcga_pan_can_atlas_2018_all&gene_list=AKT3%250AAPH1A%250ABMP7%250ACCND1%250A
CDKN2A%250ACDKN2B%250AE2F5%250AEIF4EBP1%250AERBB2%250AFGFR1%250AHEY1%250
AIRF2BP2%250AKAT6A%250AMAP3K3%250AMCL1%250AMDM4%250AMMP16%250AMYC%250AN
CSTN%250ANOTCH2%250APAK1%250APARP1%250APSEN2%250APTEN%250APTK2%250ARAB2
5%250ARIT1%250ARNF43%250ARPS6KB1%250ARPS6KB2%250ARYR2%250ASPOP%250ATGFB2
%250AUSH2A%250AZNF217&geneset_list=%20&tab_index=tab_visualize&Action=Submit&genetic_prof
ile_ids_PROFILE_COPY_NUMBER_ALTERATION=luad_tcga_pan_can_atlas_2018_gistic

Liver cancer:
https://www.cbioportal.org/results/download?cancer_study_list=lihc_tcga_pan_can_atlas_2018&Z_SCOR
E_THRESHOLD=2.0&RPPA_SCORE_THRESHOLD=2.0&data_priority=0&profileFilter=0&case_set_id=li

hc_tcga_pan_can_atlas_2018_all&gene_list=AKT3%250AAPH1A%250ABMP7%250ACCND1%250ACDKN2A%250ACDKN2B%250AE2F5%250AEIF4EBP1%250AERBB2%250AFGFR1%250AHEY1%250AIRF2BP2%250AKAT6A%250AMAP3K3%250AMCL1%250AMDM4%250AMMP16%250AMYC%250ANCSTN%250ANOTCH2%250APAK1%250APARP1%250APSEN2%250APTEN%250APTK2%250ARAB25%250ARIT1%250ARNF43%250ARPS6KB1%250ARPS6KB2%250ARYR2%250ASPOP%250ATGFB2%250AUSH2A%250AZNF217&geneset_list=%20&tab_index=tab_visualize&Action=Submit&genetic_profile_ids_PROFILE_COPY_NUMBER_ALTERATION=lihc_tcga_pan_can_atlas_2018_gistic

The omics data (mRNA expression, copy number alteration) used for pathway activity can be downloaded from https://www.cbioportal.org/datasets:
https://cbioportal-datahub.s3.amazonaws.com/brca_tcga_pan_can_atlas_2018.tar.gz
https://cbioportal-datahub.s3.amazonaws.com/luad_tcga_pan_can_atlas_2018.tar.gz
https://cbioportal-datahub.s3.amazonaws.com/lihc_tcga_pan_can_atlas_2018.tar.gz
