# Supplementary material for: Genetic mutation and biological pathway prediction based on whole slide images in breast carcinoma using deep learning
Source: NPJ Precis Oncol. 2021 Sep 23;5:87. doi: 10.1038/s41698-021-00225-9 (PMC8460699; doi:10.1038/s41698-021-00225-9)
Supplement: Supplementary file 2 — Reporting Summary [file 41698_2021_225_MOESM2_ESM.pdf]

nature research

Corresponding author(s): Dimitris Metaxas

Last updated by author(s): Mar 18, 2021

Reporting Summary

Nature Research wishes to improve the reproducibility of the work that we publish. This form provides structure for consistency and transparency in reporting. For further information on Nature Research policies, see our Editorial Policies and the Editorial Policy Checklist.

Statistics

For all statistical analyses, confirm that the following items are present in the figure legend, table legend, main text, or Methods section.

n/a	Confirmed	
☐	☒	The exact sample size (n) for each experimental group/condition, given as a discrete number and unit of measurement
☐	☒	A statement on whether measurements were taken from distinct samples or whether the same sample was measured repeatedly
☒	☐	The statistical test(s) used AND whether they are one- or two-sided<br>*Only common tests should be described solely by name; describe more complex techniques in the Methods section.*
☐	☒	A description of all covariates tested
☐	☒	A description of any assumptions or corrections, such as tests of normality and adjustment for multiple comparisons
☐	☒	A full description of the statistical parameters including central tendency (e.g. means) or other basic estimates (e.g. regression coefficient) AND variation (e.g. standard deviation) or associated estimates of uncertainty (e.g. confidence intervals)
☒	☐	For null hypothesis testing, the test statistic (e.g. F, t, r) with confidence intervals, effect sizes, degrees of freedom and P value noted<br>*Give P values as exact values whenever suitable.*
☒	☐	For Bayesian analysis, information on the choice of priors and Markov chain Monte Carlo settings
☒	☐	For hierarchical and complex designs, identification of the appropriate level for tests and full reporting of outcomes
☒	☐	Estimates of effect sizes (e.g. Cohen's d, Pearson's r), indicating how they were calculated

Our web collection on statistics for biologists contains articles on many of the points above.

Software and code

Policy information about availability of computer code

Data collection	The WSI data were collected from Genomic Data Commons data portal (https://portal.gdc.cancer.gov/) and the other molecular data were obtained from the cBioPortal (https://www.cbioportal.org/). The gdc-client's version is 1.5.0.
Data analysis	The codes support this work is at https://github.com/huiqu18/GeneMutationFromHE/tree/main/code_data_processing<br>The used libraries include:Python (version 3.6.8), Openslide (version 3.4.1), Openslide-python (version 1.1.0) Numpy (version 1.16.4), Pandas (version 0.25.0), Scikit-image (version 0.15.0), Scikit-learn (version 0.21.3), Pillow (version 6.1.0), PyTorch (version 1.0.0), Torchvision (version 0.3.0), h5py (version 2.9.0), matplotlib (version 2.2.2), tqdm (version 4.32.1), GIMP (version 2.10.2)

For manuscripts utilizing custom algorithms or software that are central to the research but not yet described in published literature, software must be made available to editors and reviewers. We strongly encourage code deposition in a community repository (e.g. GitHub). See the Nature Research guidelines for submitting code & software for further information.

Data

Policy information about availability of data

All manuscripts must include a data availability statement. This statement should provide the following information, where applicable:

- Accession codes, unique identifiers, or web links for publicly available datasets
- A list of figures that have associated raw data
- A description of any restrictions on data availability

The whole slide images used in this study are publicly available through the Genomic Data Commons data portal (https://portal.gdc.cancer.gov/). The omics data (mutation, copy number alteration and mRNA expression data) are publicly available through cBioPortal (https://www.cbioportal.org/), and the download links are provided in the supplemental materials.

Field-specific reporting

Please select the one below that is the best fit for your research. If you are not sure, read the appropriate sections before making your selection.

☒ Life sciences ☐ Behavioural & social sciences ☐ Ecological, evolutionary & environmental sciences

For a reference copy of the document with all sections, see nature.com/documents/nr-reporting-summary-flat.pdf

Life sciences study design

All studies must disclose on these points even when the disclosure is negative.

Sample size	We collected 659 patients with breast invasive carcinoma from The Cancer Genome Atlas (TCGA)17. Data inclusion criteria for each patient contain: 1) hematoxylin and eosin (H&E) stained histopathology whole-slide image (WSI), 2) mutational data with the point mutation status of 18 driver genes and copy number alteration (CNA) of 35 genes (see Methods), and 3) omics data with the mRNA expression data and CNA data of all genes. The WSI data were downloaded from Genomic Data Commons data portal (https://portal.gdc.cancer.gov/) and the other molecular data were obtained from the cBioPortal (https://www.cbioportal.org/). In addition, we collected 350 patients with lung adenocarcinoma from TCGA-LUAD cohort and 316 patients with liver hepatocellular carcinoma from TCGA-LIHC cohort to validate our method following the same data inclusion criteria.
Data exclusions	Patient samples with missing CNA and mutation data were removed from analysis. Patient samples with poor quality of pathological scans were removed from analysis.
Replication	We provided a complete list of patients from TCGA to allow the data available to the community. Also, we provided Github code repository with data processing and model analysis.
Randomization	The 659 cases in the TCGA-BRCA dataset were randomly partitioned into training, validation and test sets based on 70%, 15%, and 15% ratios respectively. This is commonly used in machine learning to evaluate prediction performance. Also, for TCGA-LUAD, TCGA-LIHC datasets, each dataset is split into training and testing sets with 20% and 80% ratios, where the training set is utilized to fine-tune the developed models trained from the breast cancer data.
Blinding	Blinding is not relevant to the study, since we are applying deep learning models to predict the mutation states with proper training/testing designs.

Reporting for specific materials, systems and methods

We require information from authors about some types of materials, experimental systems and methods used in many studies. Here, indicate whether each material, system or method listed is relevant to your study. If you are not sure if a list item applies to your research, read the appropriate section before selecting a response.

Materials & experimental systems

n/a	Involved in the study	
☒	☐	Antibodies
☒	☐	Eukaryotic cell lines
☒	☐	Palaeontology and archaeology
☒	☐	Animals and other organisms
☒	☐	Human research participants
☒	☐	Clinical data
☒	☐	Dual use research of concern

Methods

n/a	Involved in the study	
☒	☐	ChIP-seq
☒	☐	Flow cytometry
☒	☐	MRI-based neuroimaging
